# SRSF3-TRIM28-MDC1 prevents DNA damage caused by R-loops in fatty liver disease in mice

**DOI:** 10.1172/jci.insight.188629

**Published:** 2026-01-09

**Authors:** Panyisha Wu, Manasi Das, Yanting Wang, Yichun Ji, Yuli Wu, Deepak Kumar, Lily J. Jih, Nicholas J.G. Webster

**Affiliations:** 1 A San Diego Healthcare System, San Diego, California, USA.; 2 Department of Medicine, Division of Endocrinology and Metabolism, University of California San Diego, La Jolla, California, USA.; 3 Department of Gastroenterology, The Second Affiliated Hospital of Zhejiang University School of Medicine, Hangzhou, China.; 4 Department of Pathology and; 5 Moores Cancer Center, University of California San Diego, La Jolla, Califonria, USA.

**Keywords:** Endocrinology, Hepatology, DNA repair, Liver cancer, RNA processing

## Abstract

Serine-rich splicing factor 3 (SRSF3) is crucial for the metabolic functions of the liver. The genetic deletion of SRSF3 in mouse hepatocytes impairs hepatic lipid and glucose metabolism and leads to fibrosis and formation of hepatocellular adenoma that progresses to hepatocellular carcinoma. SRSF3 protein is proteosomally degraded in metabolic-dysfunction associated fatty liver disease (MAFLD) and metabolic-dysfunction-associated steatohepatitis (MASH). We show here that depleting SRSF3 protein in hepatocytes promoted R-loop accumulation and increased DNA damage in the liver. Prevention of SRSF3 degradation in vivo protected hepatocytes from DNA double-strand breaks in mice with MASH. This protection extended to other DNA-damaging agents such as camptothecin, palmitic acid, or hydrogen peroxide when tested on HepG2 cells in vitro. SRSF3 interacted with TRIM28 and MDC1, which are components of the ATM DNA-damage repair complex, and knockdown of any of these 3 proteins reduced the expression of the other 2 proteins, suggesting they form a functional complex. Lastly, by preventing degradation of SRSF3, we were able to reduce tumors in a diethyl-nitrosamine–induced (DEN-induced) model of cirrhotic HCC. These findings suggest that maintenance of SRSF3 protein stability is crucial for preventing DNA damage and protecting liver from early metabolic liver disease and progression to HCC.

## Abstract 2

In this paper, the authors showed that loss of a splicing factor SRSF3 causes formation of R-loops and DNA damage that ultimately leads to liver cancer.

## Graphical abstract

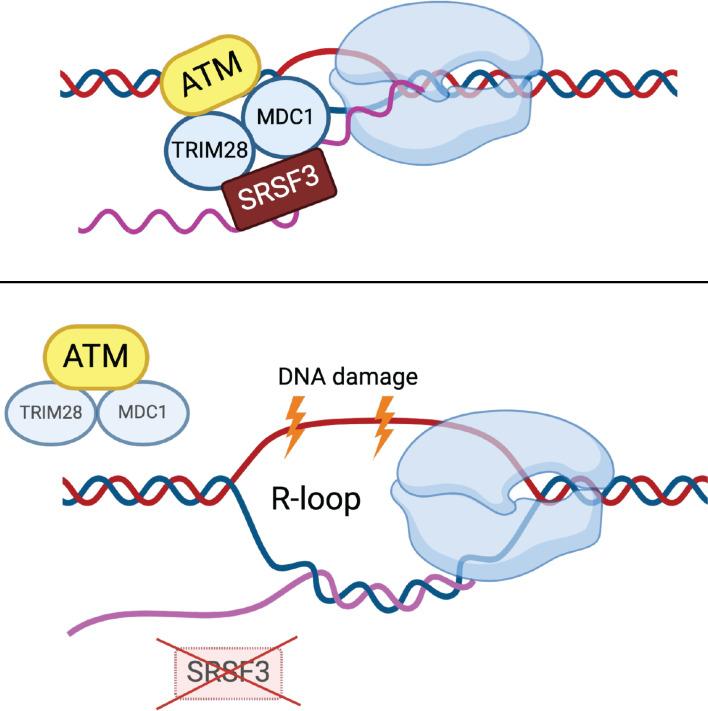

## Introduction

Metabolic dysfunction-associated fatty liver disease (MAFLD) is the leading cause of liver disease in Western countries. It is defined as the fat accumulation in the liver after the exclusion of secondary causes (1). MAFLD can progress from simple steatosis to metabolic dysfunction-associated steatohepatitis (MASH), cirrhosis, and even hepatocellular carcinoma (HCC) (2–4). Many obesity-related genes are regulated by alternative splicing, and studies have suggested that these splicing variants play a critical role in MAFLD development (5, 6). Similarly, altered splicing factor expression has been reported in patients with MAFLD and/or obesity (7). Thus, mounting evidence strongly points to an association between altered RNA splicing and liver disease.

SRSF3 is the smallest member of the SR protein family. SR proteins are a family of RNA binding proteins that are involved in alternative splicing of RNA. We have previously shown that the deletion of SRSF3 impairs splicing of selected RNA isoforms, resulting in inhibition of hepatocyte maturation, impairment of lipid and glucose metabolism, generation of endoplasmic reticulum stress, and development of HCC (8, 9). Furthermore, we have shown that SRSF3 is reduced in early-stage human liver disease and cirrhosis, and preventing SRSF3 degradation prevents MASH in mouse models (10).

There are many studies demonstrating DNA damage in fatty liver disease (11–13). MASH is a risk factor for the development of HCC, and DNA damage is elevated in MASH in mice and humans. Recent studies have suggested that splicing factors may play regulate R-loop resolution (14, 15) and prevent transcription-associated DNA damage in cancer (16). R-loops are nucleic acid structures that form at the sites of gene transcription. They form when the transcribed RNA strand base pairs with the template DNA in the transcription bubble, which prevents the reassociation of the 2 DNA strands and leaves the nontemplate DNA strand susceptible to damage (17). If R-loops are not quickly resolved, they can lead to DNA damage, genome instability, and ultimately cell death (16, 18). SRSF1 and SRSF3 have been shown to be important for maintaining genomic stability and preventing DNA damage in various cell lines by binding to RNA and destabilizing R-loops (19–23); however, the role of R-loops in the progression of fatty liver disease remains unclear (24). Therefore, in this study, we investigated the role of R-loops and DNA damage in hepatocytes due to the loss of SRSF3 in vivo.

## Results

### DNA damage in livers from MAFLD and MASH mice correlates with loss of SRSF3.

**Figure 1:**
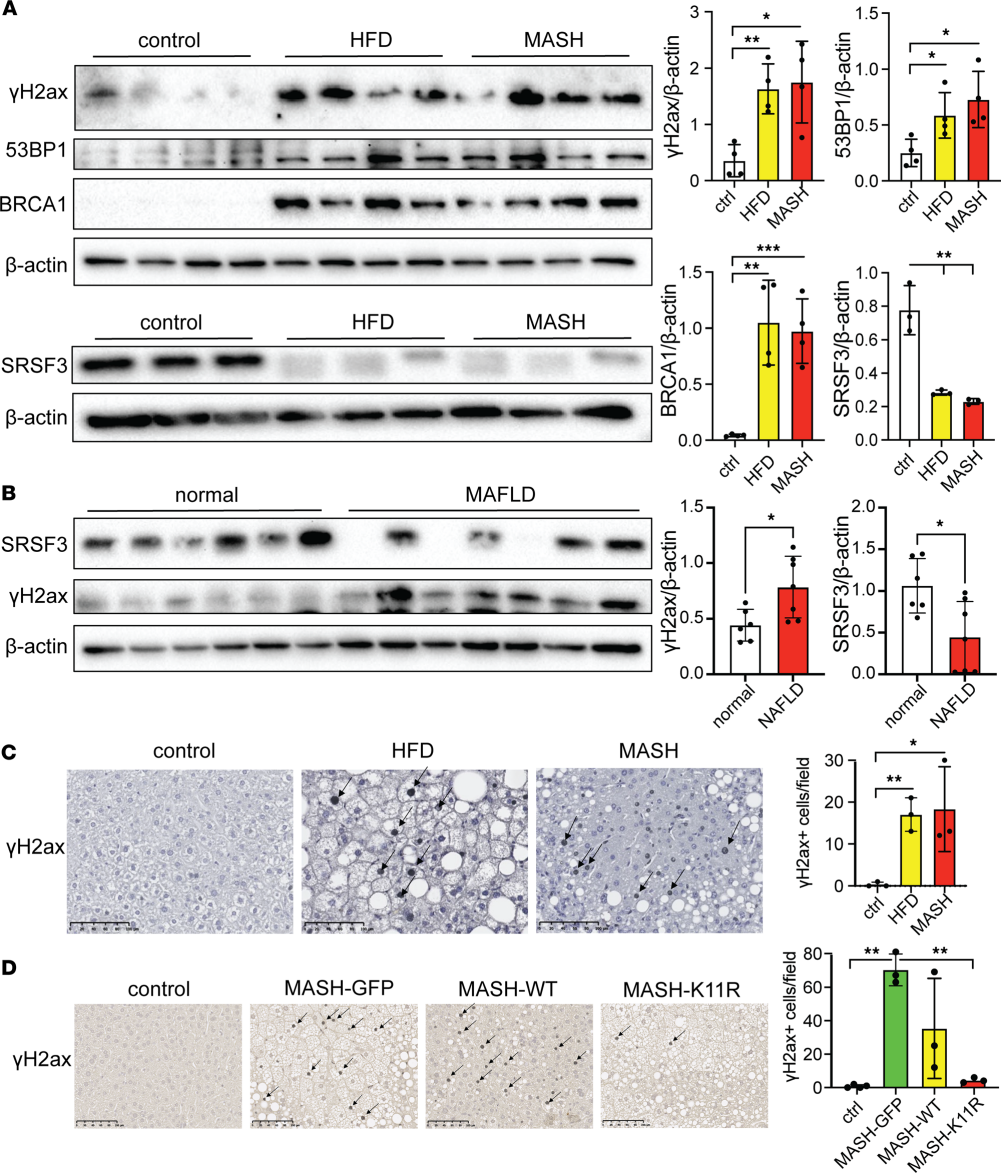
Preventing SRSF3 degradation reduces hepatocyte DNA damage in vivo. (**A**) Immunoblotting of γH2ax, 53BP1, BRCA1, and SRSF3 in livers from mice on high-fat diet (MAFLD) or Western diet (MASH), or lean mice on normal chow (control). Graph shows quantification of γH2ax, 53BP1, BRCA1, and SRSF3 protein levels normalized to β-actin (*n* = 3–4/group). Lean mice are shown in white, MAFLD mice in yellow, and MASH mice in red. (**B**) Immunoblotting of γH2ax and SRSF3 from normal or MAFLD human livers. Graph shows quantification of γH2ax and SRSF3 protein levels normalized to β-actin (*n* = 6–7/group). Normal is shown in white and MAFLD in red. (**C**) Immunohistochemical staining for γH2ax on FFPE sections from livers of lean, MAFLD, and MASH mice. Arrows indicate representative positive nuclei. Graph shows quantification of γH2ax^+^ nuclei per field (*n* = 3/group). Scale bars: 100 μm. (**D**) Immunohistochemical staining for γH2ax on FFPE liver sections lean mice on normal chow (control) or mice on Western diet infected with AAV8 expressing GFP (MASH-GFP), WT SRSF3 (MASH-WT), or the degradation-resistant K11R-mutant SRSF3 (MASH-K11R). Arrows indicate representative positive nuclei. Graph shows quantification of γH2ax^+^ nuclei/field (*n* = 3/group). Scale bars: 100 μm. All quantified results are presented as mean ± SD; *P < 0.05, **P < 0.01, ***P < 0.001 by 1-way ANOVA.

To demonstrate DNA damage in our mouse models of MAFLD and MASH, we immunoblotted liver lysates for γH2ax, a highly specific and sensitive molecular marker for DNA double-strand breaks (25). γH2ax levels by Western blot were elevated in both the MAFLD and MASH livers (Figure 1A). We also observed elevated levels of 53BP1 and BRCA1, 2 other markers of DNA damage (Figure 1A). As expected, SRSF3 expression was lower in the mouse MAFLD and MASH livers (Figure 1A). In human samples, γH2ax levels were elevated in MAFLD livers and SRSF3 levels reduced (Figure 1B) (10). We confirmed that the damage was occurring in the hepatocytes by immunostaining γH2ax on mouse liver tissue sections (Figure 1C).

Since SRSF3 is proteolytically degraded in MAFLD and MASH (10), we investigated whether preventing SRSF3 degradation could ameliorate DNA damage. We expressed the degradation-resistant mutant SRSF3-K11R using an adeno-associated virus (AAV8) in C57BL/6J mice (*n* = 3/group). Control mice received AAV8 vectors expressing green fluorescent protein (GFP) or WT SRSF3 (SRSF3-WT). The mice were placed on a Western diet for 7 weeks. We observed more γH2ax^+^ nuclei in liver sections from mice expressing GFP (MASH-GFP) or SRSF3-WT (MASH-WT) compared with lean control mice (Figure 1D), but the liver sections from the SRSF3-K11R–infected mice (MASH-K11R) showed reduced γH2ax^+^ nuclei (Figure 1D) indicating less DNA damage.

### Acute loss of SRSF3 causes DNA damage in vitro.

**Figure 2:**
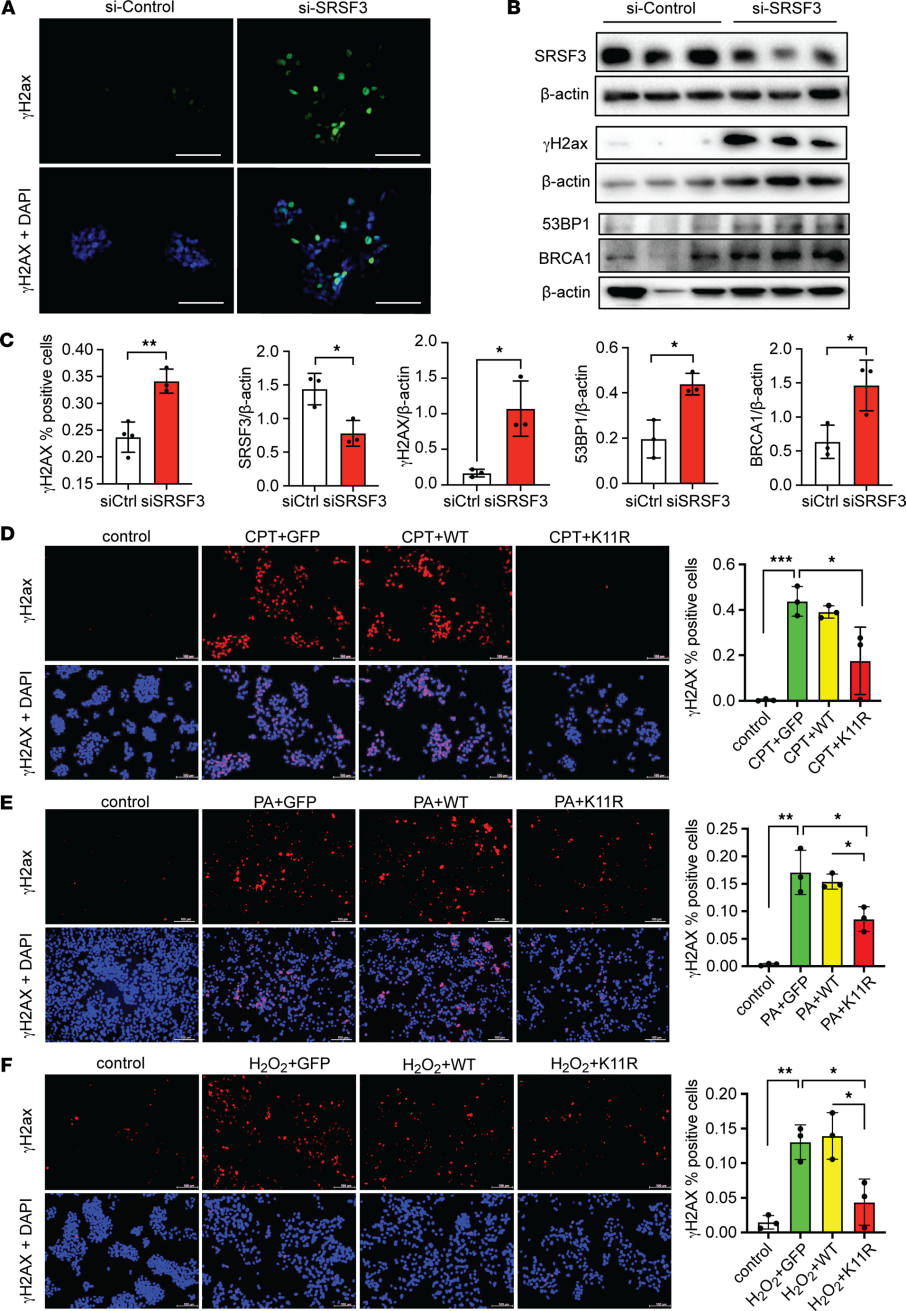
Preventing SRSF3 degradation reduces HepG2 cell DNA damage in vitro. (**A**) Immunofluorescence staining for γH2ax in HepG2 cells (green) treated with control or SRSF3 siRNA (20 nM, 48 hours). DAPI was strained to visualize the nuclei (blue). Panels show γH2AX fluorescence alone or merged with DAPI. Scale bar: 100 μm. (**B**) Immunoblotting of SRSF3, γH2ax, 53BP1, and BRCA1 from human hepatocytes with or without SRSF3 knockdown. Gels were run in parallel, and individual actin control blots are shown. (**C**) Graphs showing percentage of γH2ax^+^ nuclei by immunofluorescence (*n* = 3/group) or SRSF3, γH2ax, 53BP1, and BRCA1 protein levels normalized to β-actin by Western blot (*n* = 3/group). (**D**–**F**) HepG2 cells were infected with AAV8 expressing GFP, SRSF3-WT, SRSF3-K11R directly at MOI 500,000 for 48 hours. γH2ax was detected by immunofluorescence (red) following induction of DNA damage with 0.1 μM CPT for 1 hour (**D**), 500 μM PA for 12 hours (**E**), or 200 μM H_2_O_2_ for 1 hour (**F**). Nuclei were counterstained with DAPI (blue). Scale bars: 100 μm. Graphs show quantification of γH2ax^+^ nuclei/field by immunofluorescence (*n* = 3/group). Control is shown in white, GFP in green, WT in yellow, and K11R in red. All quantified results are presented as mean ± SD; *P < 0.05, **P < 0.01, ***P < 0.001 by 1-way ANOVA.

Having observed a correlation of SRSF3 loss and DNA damage, we wanted to test whether the loss of SRSF3 caused DNA damage. SRSF3 was knocked down using siRNA in human hepatocytes. The percentage of γH2ax^+^ cells was significantly higher in SRSF3 knockdown cells by immunofluorescence (Figure 2, A and C), which was consistent with higher expression of γH2ax, 53BP1, and BRCA1 in cell extracts (Figure 2, B and C). Similarly, γH2ax levels were elevated in extracts of hepatocytes from our SRSF3-HKO mouse (Supplemental Figure 1A; supplemental material available online with this article; https://doi.org/10.1172/jci.insight.188629DS1) (26).

We then tested whether the degradation-resistant mutant SRSF3 could prevent DNA damage due to other agents. We induced DNA damage in HepG2 cells using the topoisomerase inhibitor camptothecin (CPT; 5 μM for 1 hour [h]). As in the livers in vivo, AAV8 expression of SRSF3-K11R before CPT treatment (0.1 μM for 1 h) significantly decreased the percentage of γH2ax^+^ cells compared with cells expressing GFP or SRSF3-WT (Figure 2D). Interestingly, SRSF3 protein expression was reduced following CPT treatment (Supplemental Figure 1, B and C). We confirmed this result by preventing SRSF3 degradation using the NEDD8-activating enzyme (NAE) inhibitor (MLN4924). NAE inhibition reduced DNA damage following CPT treatment (5 μM for 1 h), by immunostaining and immunoblot (Supplemental Figure 1, D and E).

We then stressed HepG2 cells by lipid overload or hydrogen peroxide. Palmitic acid (PA) treatment caused a dose-dependent loss of SRSF3 protein (Supplemental Figure 1F) as expected (10). PA treatment increased the percentage of γH2ax^+^ nuclei in cells expressing GFP or SRSF3-WT but cells expressing SRSF3-K11R exhibited significantly fewer γH2ax^+^ nuclei (Figure 2E). The same result was observed in primary human hepatocytes (Supplemental Figure 1G). Similarly, when we treated HepG2 cells with H_2_O_2_ to cause oxidative DNA damage, we found less γH2ax^+^ nuclei in cells expressing SRSF3-K11R (Figure 2F). These results indicate that stabilization of SRSF3 prevented DNA damage in response to multiple agents both in vivo and in vitro.

### Deletion of SRSF3 leads to R-loop accumulation.

**Figure 3:**
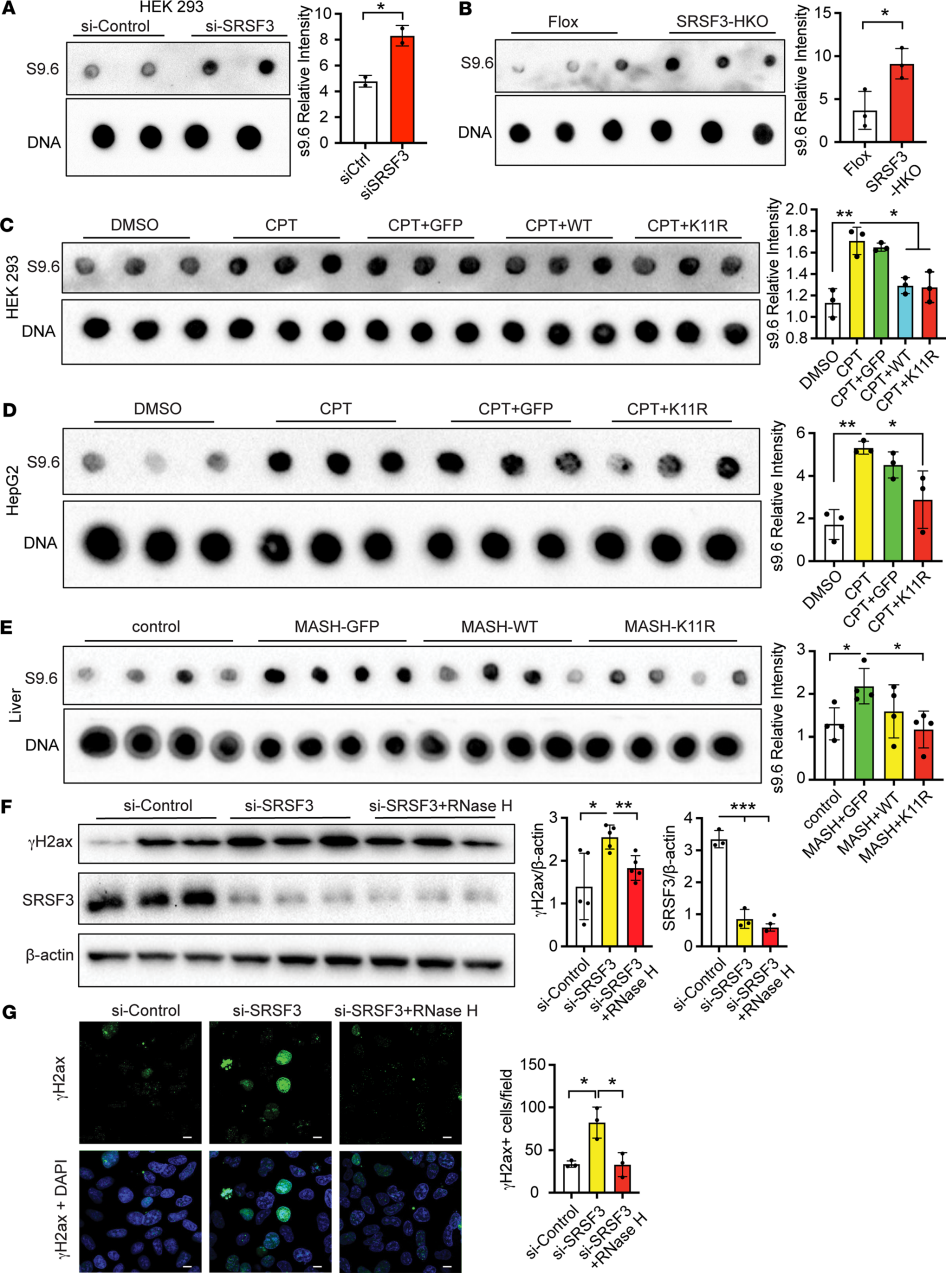
Knockdown of SRSF3 causes R-loop accumulation. (**A**) Dot blot of R-loops using antibody S9.6 in HEK293 cells treated with control siRNA (si-Control) or SRSF3 siRNA (si-SRSF3). Bots were stripped and reblotted for double-stranded DNA (dsDNA). Graph shows quantification of R-loop levels normalized to dsDNA (*n* = 2/group). (**B**) Dot blot of R-loops in genomic DNA from Flox and SRSF3-KO hepatocytes (SRSF3-HKO). Graph shows quantification of R-loop levels normalized to dsDNA (*n* = 3/group). (**C**) HEK293 cells were transfected by GFP, Flagged-SRSF3-WT, or Flagged-SRSF3-K11R plasmids for 48 hours, before being treated with 0.1 μM CPT or DMSO (vehicle control) for 1 hour and R-loops detected by dot blot. Graph shows quantification of R-loop levels normalized to dsDNA (*n* = 3/group). (**D**) HepG2 cells were infected by AAV8 expressing GFP or Flagged-SRSF3-K11R, before being treated with 0.1 μM CPT or DMSO for 1 hour, and R-loops were detected by dot blot. Graph shows quantification of R-loop levels normalized to dsDNA (*n* = 3/group). (**E**) Dot blot of R-loops in genomic DNA from livers of lean mice on normal chow (control) or mice on a Western (MASH) diet 7 weeks after infection with AAV8 expressing GFP (MASH-GFP), WT SRSF3 (MASH-WT), or the degradation-resistant K11R-mutant SRSF3 (MASH-K11R). Graph shows quantification of R-loop levels normalized to dsDNA (*n* = 3/group). (**F**) Immunoblotting of SRSF3, γH2ax from HEK293 cells with or without SRSF3 knockdown. Five units of RNase H protein were transfected in selected wells for 4 hours to digest R-loops. Graph shows quantification of SRSF3 and γH2ax protein levels normalized to β-actin (*n* = 3/group). (**G**) Immunofluorescent staining for γH2ax in HEK293 cells treated as above. DAPI was stained to visualize the nuclei. ce staining for TRIM28 and SRSF3. In all cases DAPI was used to visualize the nuclei. Scale bar: 10 μm. Original magnification, ×630. Graph shows quantification of γH2ax^+^ nuclei/field (*n* = 3/group). All quantified results are presented as mean ± SD; *P < 0.05, **P < 0.01, ***P < 0.001 by 1-way ANOVA.

Splicing factors have been associated with R-loop formation during transcription and replication (14, 15, 27), so we investigated whether loss of SRSF3 introduced more R-loops. SRSF3 was knocked down by siRNA and R-loops assessed on genomic DNA in a dot blot assay using antibody S9.6 that specifically binds to RNA-DNA hybrids. HEK293 cells were used for these experiments due to their high transfection efficiency. SRSF3 knockdown increased S9.6 immunoreactivity indicating elevated levels of R-loops when normalized to input DNA (Figure 3A). RNaseH was added to control wells to digest RNA-DNA hybrids and confirm the specificity of S9.6 binding (28), and in all cases, incubation with RNaseH eliminated S9.6 staining, confirming that staining was due to R-loops (Supplemental Figure 2, A–D). We purified genomic DNA from livers of SRSF3-HKO mice and found elevated S9.6 staining in SRSF3-HKO livers compared with Flox mice indicating more R-loops (Figure 3B).

Since blocking SRSF3 degradation prevented DNA damage, we tested whether R-loop formation was similarly suppressed. The cells were transfected with GFP, SRSF3-WT, or SRSF3-K11R expression plasmids, before being treated 48 h later with CPT for 1 h. We extracted genomic DNA and performed the S9.6 dot blot assay. The CPT or CPT+GFP groups exhibited more R-loop accumulation than control cells (Figure 3C), but the cells expressing SRSF3-K11R or SRSF3-WT showed lower R-loop levels. This experiment was repeated in HepG2 cells using AAV8 expression of GFP or SRSF3-K11R proteins. CPT treatment increased R-loops in control cells and cells expressing GFP, but expression of SRSF3-K11R cells reduced R-loop formation (Figure 3D). We then assessed R-loops in livers from SRSF3-K11R–expressing mice. R-loops were increased in control mice on the Western diet and in mice expressing GFP, but livers from mice expressing SRSF3-K11R showed reduced R-loop accumulation (Figure 3E). These results show a correlation between R-loop formation and DNA damage. To directly test whether the DNA damage due to the loss of SRSF3 is a result of R-loop accumulation, we knocked down SRSF3 by siRNA in HEK293 cells, and we then transfected RNaseH protein into the cells to eliminate R-loops. DNA damage was elevated with SRSF3 knockdown, as expected, but decreased after RNaseH treatment (Figure 3, F and G), indicating that the DNA damage induced by SRSF3 knockdown was caused by elevated R-loops.

**Figure 4:**
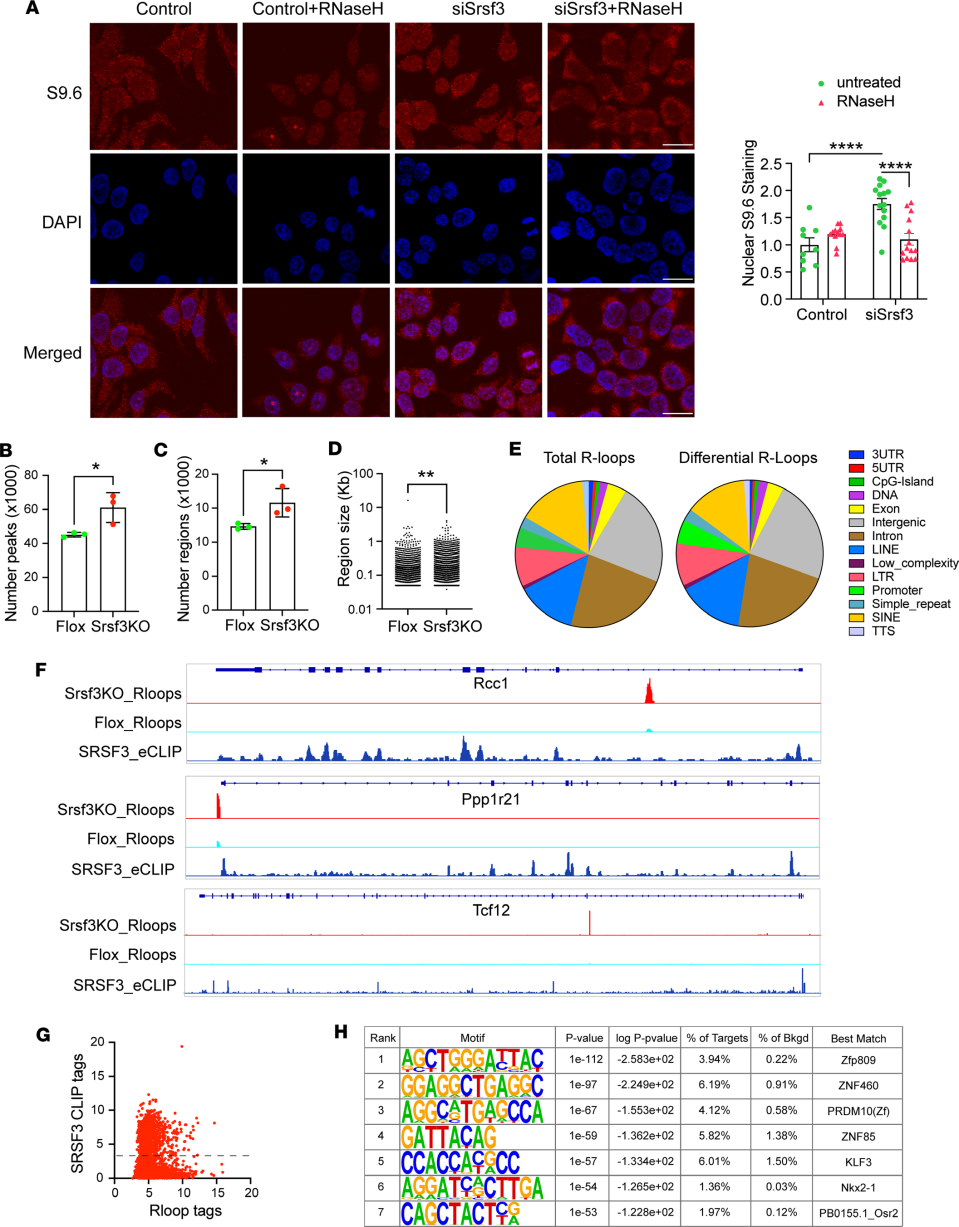
Spatial and genomic effects of SRSF3 on R-loops. (**A**) Immunofluorescence staining for R-loops with antibody S9.6 in HEK293 cells with siSrsf3 knockdown. In some samples, cells were treated with RNASEH1 before S9.6 staining to digest RNA-DNA hybrids. Nuclei were counter stained with DAPI and S9.6 staining was quantified specifically in nuclei. Scale bars: 10 μm. (**B**) Genomic R-loops were profiled in primary hepatocytes with acute deletion of SRSF3 using Cut&TAG and short-read sequencing. Reads were aligned to the mm10 genome and peaks assessed using the HOMER suite. Graph shows the total number of peaks in the control and KO hepatocytes, compared by 2-tailed *t* test. (**C**) Adjacent peaks were merged into larger R-loop regions. Graph shows total number of R-lop regions in control and KO hepatocytes. (**D**) The size of the R-loop regions was quantified. The KO hepatocytes showed an increase in R-loom size by nonparametric Kolmogorov-Smirnov test. (**E**) R-loops were annotated to known genomic features. The left pie chart shows the proportion of total R-loops assigned to different genomic features. The right pie chart shows the distribution of R-loops that were different between control and KO hepatocytes. (**F**) Three representative genes (Rcc1, Ppp1r21, and Tcf12) that showed differential R-loops in the KO hepatocytes. Gene structure is shown at top with KO and control R-loops and SRSF3 CLIP profile below. (**G**) The scatter plot shows the correlation of SRSF3 CLIP tags at sites that have differential R-loop peaks. Dotted line indicates separation of peaks into those with background levels of CLIP tags versus peaks with associated SRSF3 binding. (**H**) Motif analysis on the differential R-loops. Chart shows enriched de novo motifs with enrichment *P* value, percent of targets and background containing the motif, and best match to known factors. *P < 0.05, **P < 0.01, ****P < 0.0001. Enrichment probabilities calculated using cumulative hypergeometric distribution.

Since antibody S9.6 can also recognize cytoplasmic double-stranded RNA, we confirmed the presence of R-loops in the nucleus by immunofluorescence imaging. In control HEK293 cells, S9.6 staining was predominantly cytoplasmic and was not sensitive to RNaseH treatment. Knockdown of SRSF3 increased nuclear staining for R-loops that was eliminated by RNaseH (Figure 4A). To obtain a more quantitative assessment of R-loops and their connection to SRSF3, we performed whole-genome R-loop profiling using a Cut&TAG approach in mouse hepatocytes with acute deletion of SRSF3. KO of SRSF3 increased the number of individual R-loop peaks (Figure 4B) and increased the number of larger R-loop regions when adjacent peaks were merged (Figure 4C). The size of the R-loop regions was also significantly increased (Figure 4D). When we annotated the peaks with known genomic features, the majority of R-loops were found in intergenic or intronic regions, or LINE and SINE elements (Figure 4E and Supplemental Table 1). Although similar distribution was found for the R-loops that were differentially altered between control and KO hepatocytes, closer examination revealed that R-loops were proportionally increased in exons, 5′UTRs, intergenic regions, and LINE elements and were decreased in SINE elements (Supplemental Table 1). We compared R-loop profiles with SRSF3 binding profiles by eCLIP that we have previously published (29). Intronic, exonic, and promoter R-loops were frequently found adjacent to exons harboring SRSF3 binding sites (Figure 4F). We mapped SRSF3 binding sites to the R-loops that were differentially altered between control and KO hepatocytes; 281 R-loops of 544 (52%) had an SRSF3 binding site within 100 Kb, and 117 of 544 (22%) had a site within 10 Kb. Plotting the density of SRSF3 CLIP tags against all R-loop tags revealed that 68% (2,240 of 3,298) R-loop tags also coincided with SRSF3 tags (Figure 4G). We also performed a motif analysis on the R-loops. No known motifs were enriched in R-loops; however, several de novo motifs were enriched, although each was found in < 10% of the R-loops (Figure 4H). Comparing these to known motifs, the top 4 motifs had best matches to zinc finger protein binding sites.

### SRSF3 interacts with DNA damage response proteins TRIM28 and MDC1.

**Figure 5:**
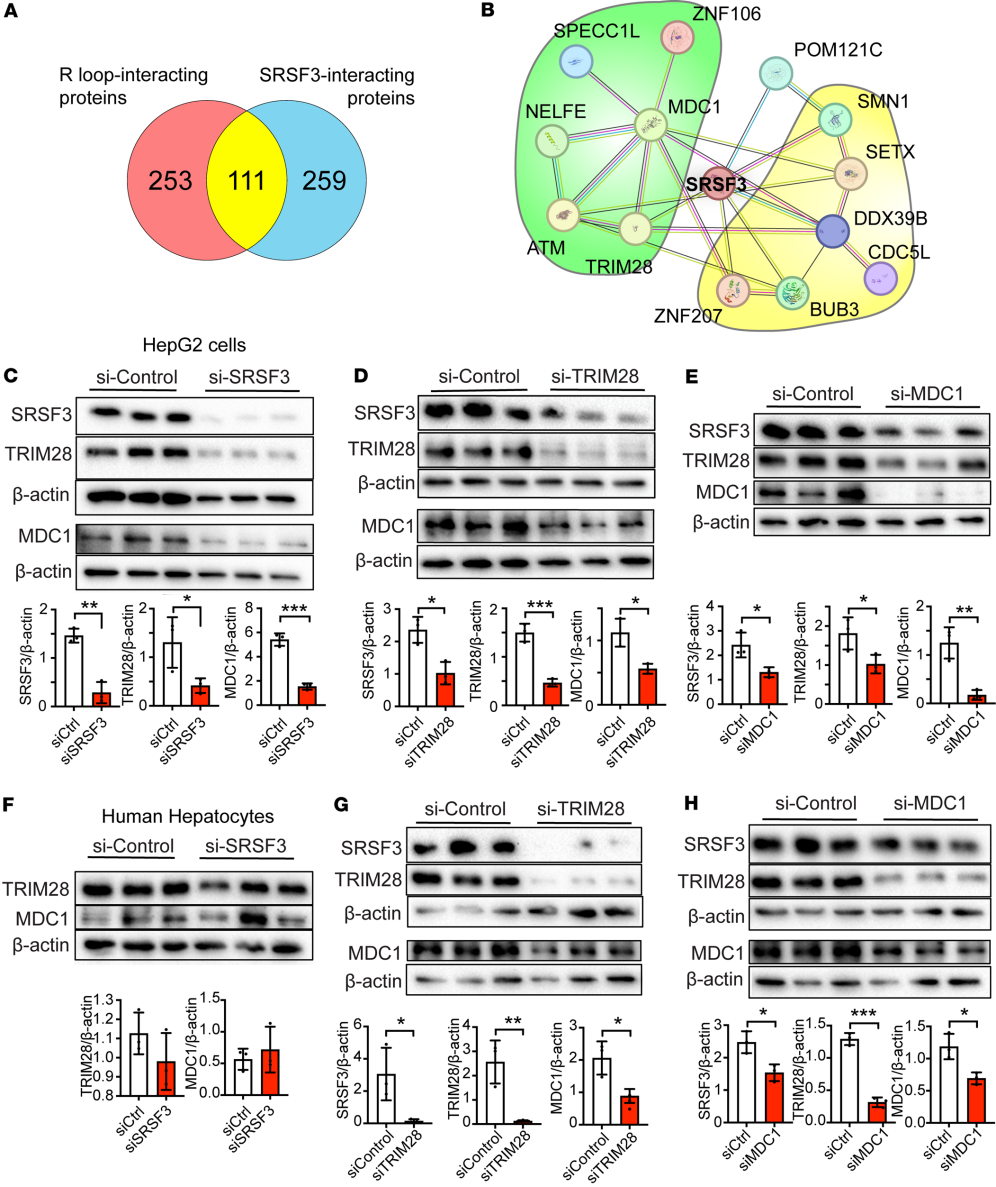
SRSF3 interacts with TRIM28 and MDC1. (**A**) Overlap of SRSF3-interacting proteins and R-loop–interacting proteins from MS data. (**B**) STRING protein-protein interaction network of proteins involved in DNA repair (green) and R-loop resolution (yellow) with SRSF3. (**C**–**E**) Immunoblots of SRSF3, TRIM28, and MDC1 proteins in HepG2 cells transfected with siRNA to SRSF3, TRIM28, or MDC1 (40 nM, 48 hours). Gels were run in parallel, and individual actin control blots are shown. Graph shows quantification of protein levels normalized to β-actin (*n* = 3/group). (**F**–**H**) Immunoblots of SRSF3, TRIM28, and MDC1 proteins in primary human hepatocytes transfected with siRNA to SRSF3, TRIM28, or MDC1 (20 nM, 48 hours). SRSF3 knockdown efficiency for **F** is shown in Figure 2B. Graph shows quantification of protein levels normalized to β-actin (*n* = 3/group).

To understand how loss of SRSF3 might cause R-loops and DNA damage, we performed affinity purification of SRSF3 from primary mouse hepatocytes and analyzed the associated proteins by LC-MS/MS (Supplemental Table 2). We compared the precipitated proteins to a dataset of proteins that interact with R-loops by cross-linking (30) and observed a striking 30% overlap of the 2 datasets (Figure 5A). We searched the STRING database for these 111 common proteins and created a protein-protein interaction network (Supplemental Figure 3A). The resulting network showed a dense cluster of ribosomal-associated proteins and another cluster of proteins involved in DNA repair. We then queried whether SRSF3 interacts with proteins with known roles in DNA repair or R-loop resolution (Figure 5B). The top 3 overall SRSF3-interacting proteins were mediator of DNA damage checkpoint 1 (MDC1), tripartite motif containing 28 (TRIM28), and Cytospin-A (SPECC1L), which are all components of the ATM DNA damage response complex. MDC1 is recruited to sites of DNA damage sites through interaction with the MRN complex, ATM, and γH2ax, allowing γH2ax phosphorylation to spread over a larger chromatin domain around DNA break sites (31); TRIM28 is an E3-ligase that is recruited to the sites of DNA damage by the ATM kinase (32); and Cytospin-A is a coiled-coil domain protein associated with actin cytoskeleton and microtubules that interacts with MDC1 (33). To confirm the interaction, we overexpressed Flag-tagged SRSF3 (Flag-SRSF3-WT or Flag-SRSF3-K11R) and HA-tagged TRIM28 (HA-TRIM28) by plasmid transfection in HEK293 cells. Cell extracts were immunoprecipitated with an anti-Flag antibody before being immunoblotted for HA or TRIM28. We were able to detect HA-reactive and TRIM28-reactive proteins precipitated with both SRSF3-WT and SRSF3-K11R (Supplemental Figure 3, B and C), confirming that SRSF3 interacted with TRIM28. The coprecipitated proteins showed multiple bands suggesting possible posttranscriptional modification of the TRIM28 protein in the complex. We also confirmed the interaction of endogenous SRSF3, TRIM28, and MDC1 proteins in mouse hepatocytes and human HepG2 cells (Supplemental Figure 3, D and E).

**Figure 6:**
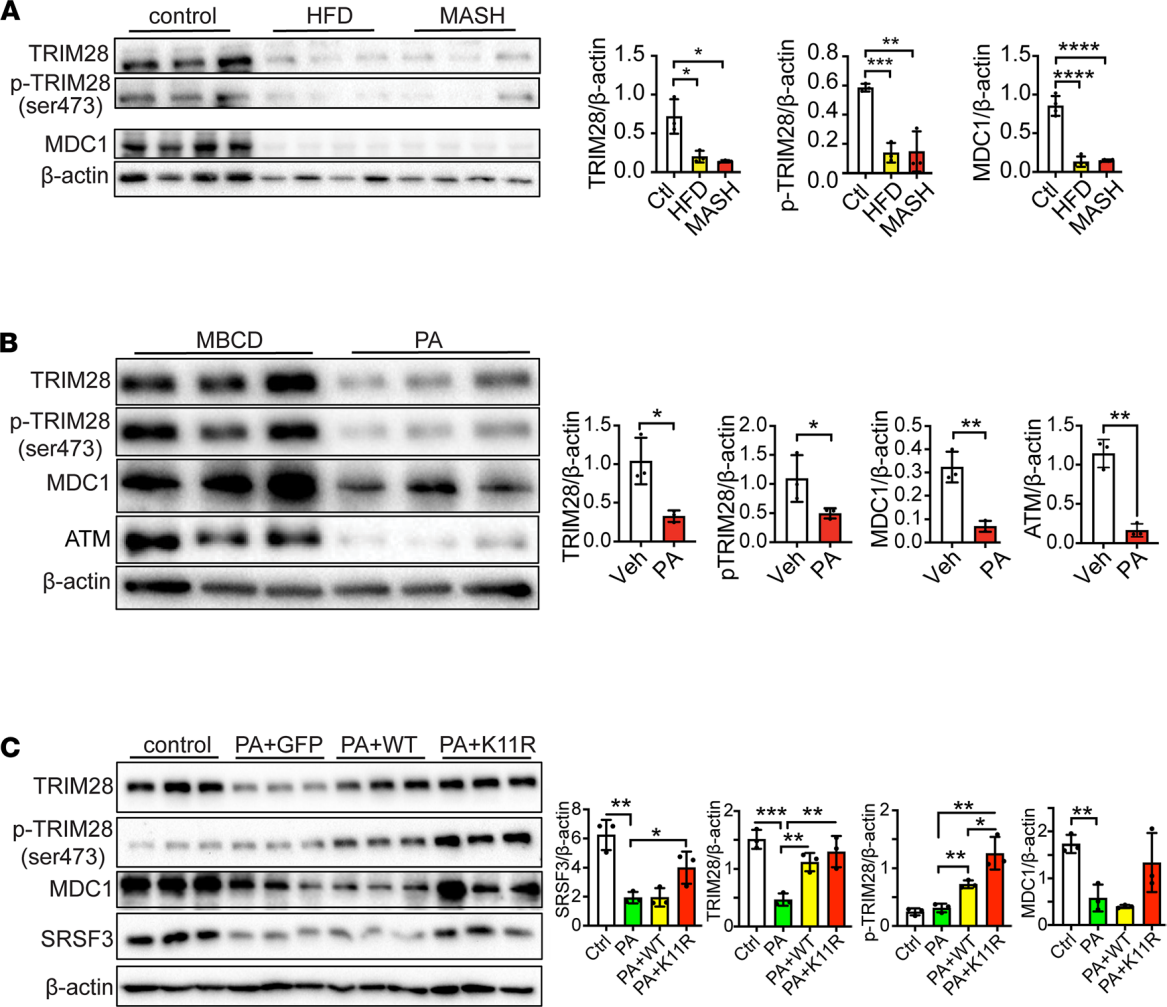
TRIM28 and MDC1 proteins are reduced by lipid overload. (**A**) Immunoblots for TRIM28, TRIM28(pSer473), and MDC1 in hepatocytes from mice on high-fat diet (MAFLD), Western diet (MASH), or normal chow (control). Actin loading control for TRIM28 is same as in Figure 1A for SRSF3. Graph shows quantification of protein levels normalized to β-actin (*n* = 3–4/group). Lean mice are shown in white, MAFLD mice in yellow, and MASH mice in red. (**B**) Immunoblots of TRIM28, TRIM28(pSer473), MDC1, and ATM from HepG2 cells treated with methyl-β-cyclodextrin (MBCD; 1 mM) as control or palmitic acid (500 μM) complexed to MBCD (1 mM) for 12 hours. Graph shows quantification of protein levels normalized to β-actin (*n* = 3/group). (**C**) Immunoblots of TRIM28, TRIM28(pSer473), MDC1, and SRSF3 in HepG2 cells infected with AAV8 expressing GFP, SRSF3-WT, SRSF3-K11R (MOI 500,000 for 48 hours) followed by 500 μM PA treatment for 12 hours. Graph shows quantification of protein levels normalized to β-actin (*n* = 3/group). Control group is shown in white, GFP in green, WT in yellow, and K11R in red. All quantified results are presented as mean ± SD; *P < 0.05, **P < 0.01, ***P < 0.001, ****P < 0.0001 by 1-way ANOVA.

Since TRIM28 and MDC1 associate with the ATM DNA repair complex, we tested whether the observed increase in γH2ax staining is due to ATM activity. Inhibition of ATM prevented the increase in γH2ax (Supplemental Figure 4A), confirming the involvement of ATM-mediated DNA repair. We then tested the effect of knockdown of SRSF3, TRIM28, or MDC1 on the stability of the other 2 proteins in HepG2 cells and primary human hepatocytes. In HepG2 cells, knockdown of either SRSF3, TRIM28, or MDC1 decreased the levels of the other 2 proteins (Figure 5, C–E) without changes in their mRNA levels (Supplemental Figure 4C). In contrast, in primary human hepatocytes, knockdown of SRSF3 did not alter TRIM28 or MDC1, but knockdown of either TRIM28 or MDC1 reduced the levels of all 3 proteins (Figure 5, F–H). In all cases, the mRNA levels did not change (Supplemental Figure 4D),suggesting posttranscriptional regulation. These results suggest that SRSF3, TRIM28, and MDC1 form a stable complex. Consistent with this finding, TRIM28 and MDC1 were significantly decreased in livers from MAFLD and MASH mice that have low levels of SRSF3 (Figure 6A).

TRIM28 is subject to posttranslational modification. Phosphorylation of TRIM28 on Ser473 is mediated by Chk1/2 (34–37), so we assessed phosphorylation of TRIM28 and found that TRIM28(pSer473) was decreased in MAFLD and MASH livers (Figure 6A). PA treatment of HepG2 cells also suppressed TRIM28(pSer4730) and decreased expression of TRIM28, MDC1, and ATM (Figure 6B). Furthermore, the decline of ATM protein level was also observed in HepG2 cells treated with stearic acid or linoleic acid (Supplemental Figure 4B). We then tested whether expression of the degradation-resistant SRSF3-K11R mutant would prevent the loss of this complex. PA suppressed TRIM28 and MDC1 expression along with SRSF3 in cells expressing GFP but had no effect in cells expressing SRSF3-K11R (Figure 6C). The results further confirmed that stress-induced degradation of SRSF3 reduced the DNA damage repair proteins TRIM28 and MDC1.

**Figure 7:**
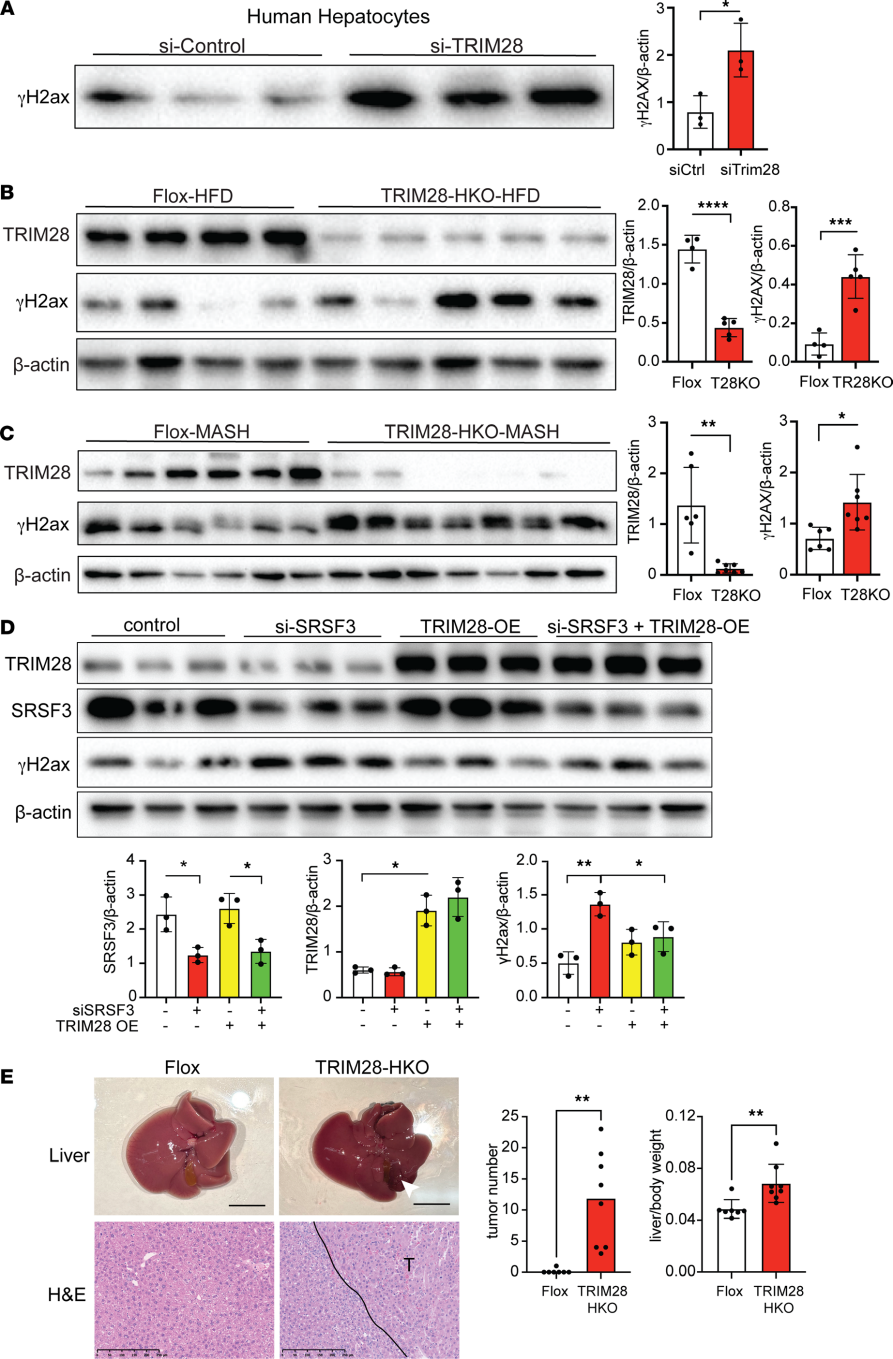
Loss of TRIM28 causes DNA damage. (**A**) Immunoblots of γH2ax in primary human hepatocytes treated with control and TRIM28 siRNA. Graph shows quantification of γH2ax protein levels normalized to β-actin (shown for MDC1 in Figure 5G) (*n* = 3/group). (**B**) Immunoblots of γH2ax and TRIM28 in hepatocytes from flox mice and TRIM28-HKO mice on high-fat diet (MAFLD) for 16 weeks. Graph shows quantification of protein levels normalized to β-actin (*n* = 4–5/group). Flox mice are shown in white and TRIM28-HKO mice in red. (**C**) Immunoblots of γH2ax and TRIM28 in hepatocytes from Flox mice and TRIM28-HKO mice on Western diet (MASH) for 12 weeks. Graph shows quantification of protein levels normalized to β-actin (*n* = 6-7/group). Flox mice are shown in white and TRIM28-HKO mice in red. 33. (**D**) Immunoblots of γH2ax, TRIM28, and SRSF3 in HEK cells transfected with SRSF3 siRNA or/and HA-TRIM28 plasmid. Graph shows quantification of protein levels normalized to β-actin (n=3/group). (**E**) Whole liver images and H&E-stained sections from 15- to 18-month Flox mice and TRIM28-HKO mice. Black line indicates tumor-normal border. Scale bar for liver pictures: 1 cm. Scale bar for sections: 250 μm. Graphs show total tumor number and liver to body weight ratio (*n* = 7-8/group). Flox mice are shown in white and TRIM28-HKO mice in red. All quantified results are presented as mean ± SD; *P < 0.05, **P < 0.01, ***P < 0.001, ****P < 0.0001 by 2-tailed *t* test or 1-way ANOVA.

Given the interaction of SRSF3 and TRIM28, we proceeded to check DNA damage in TRIM28–knocked-down human hepatocytes. γH2ax protein levels were elevated in TRIM28–knocked-down hepatocytes (Figure 7A). The result was confirmed in hepatocyte-specific TRIM28-KO mice (TRIM28-HKO). Livers from male mice showed the expected reduction in TRIM28 but also elevated γH2ax in livers from KO mice with MAFLD or MASH (Figure 7, B and C). Similar increases were seen in female livers (Supplemental Figure 5). To test if the DNA damage seen with loss of SRSF3 was due to concomitant loss of TRIM28, we overexpressed TRIM28 in HepG2 cells with SRSF3 knockdown. Overexpression of TRIM28 reduced the levels of γH2ax (Figure 7D), indicating that part of the DNA damage caused by loss of SRSF3 may be explained by loss of TRIM28-dependent DNA repair. Consistent with increased DNA damage, loss of TRIM28 predisposed both male and female mice to the development of HCC with aging and increased the liver/body weight ratio (Figure 7E) as has been reported in other hepatocyte-specific TRIM28/KAP1 KO mice (38–40) and the SRSF3-HKO mice that we have reported previously (9).

To assess relevance of these proteins for human HCC, we analyzed the TCGA HCC database for expression of TRIM28, MDC1, SRSF3, and ATM and their association with overall survival. Expression of TRIM28 and MDC1 are increased in HCC, whereas SRSF3 is not altered; ATM is slightly lower, and higher expression of either TRIM28, MDC1, or SRSF3 — but not ATM — correlates with worse survival (Supplemental Figure 6A). We extended this analysis to include proteins involved in R-loop resolution. Expression of RNASEH1, DDX39, BUB3, and ZNF207 are all increased in HCC, and higher levels correlate with worse prognosis (Supplemental Figure 6B).

### SRSF3 colocalizes with phosphorylated TRIM28 during DNA damage.

**Figure 8:**
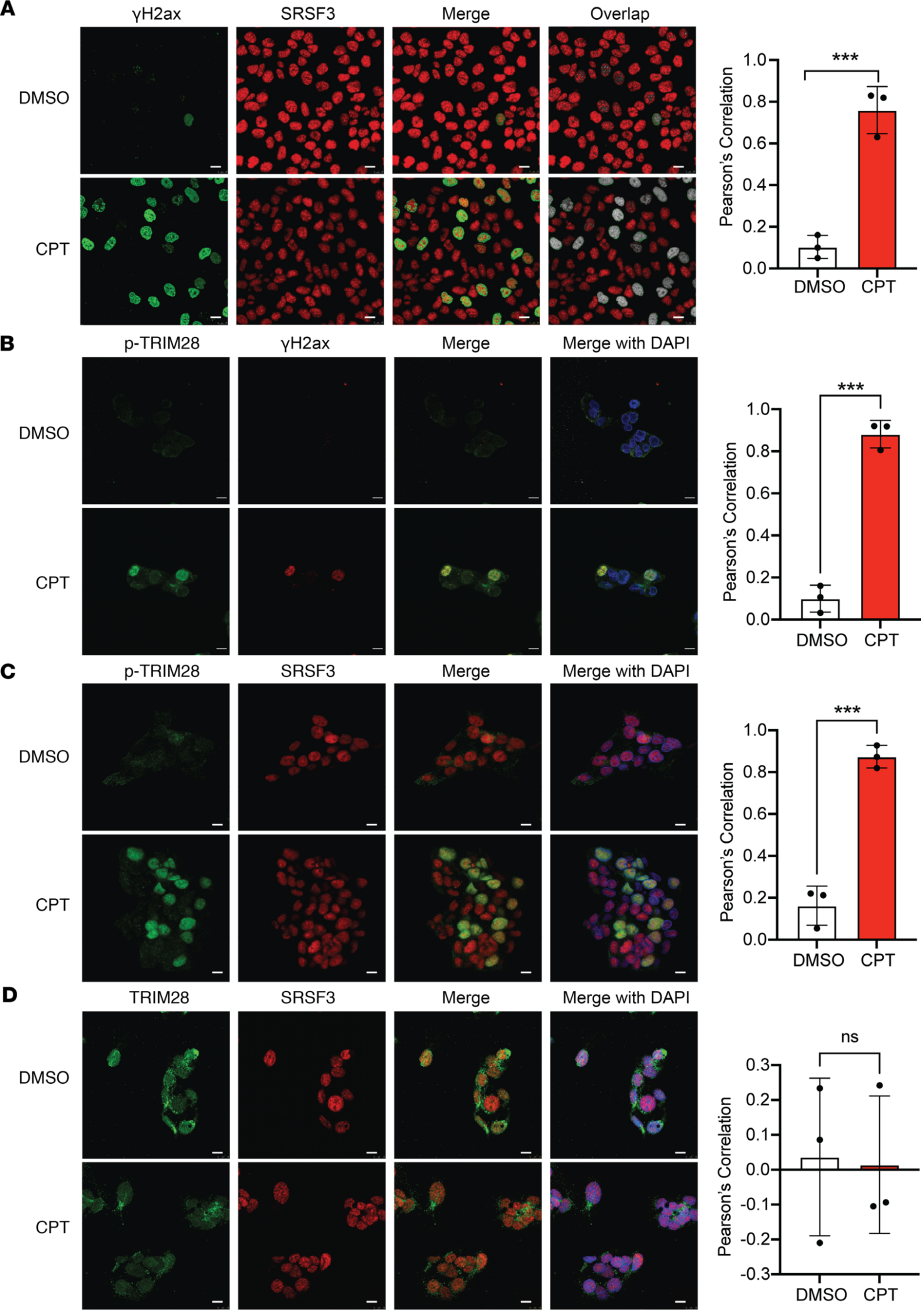
SRSF3 and TRIM28(pSer824) colocalize to sites of DNA damage. HepG2 cells were treated with DMSO or CPT (5 μM) for 1 hour. (**A**) Immunofluorescence staining for γH2ax and SRSF3. (**B**) Immunofluorescence staining for TRIM28(pSer824) and γH2ax. (**C**) Immunofluorescence staining for TRIM28(pSer824) and SRSF3. (**D**) Immunofluorescence staining for TRIM28 and SRSF3. In all cases DAPI was used to visualize the nuclei. Scale bars: 10 μm. Graph shows Pearson’s correlation coefficient (*n* = 3/group) derived from overlap image. DMSO (control) group is shown in white and CPT group shown in red. All quantified results are presented as mean ± SD; ***P < 0.001 by 2-tailed *t* test.

Since SRSF3-K11R prevented DNA damage, we analyzed the colocalization of SRSF3 to sites of DNA damage and found that SRSF3 localization strongly correlated with γH2ax staining in CPT-treated cells but not in control cells (Figure 8A). TRIM28(pSer473) phosphorylation was strongly induced in nucleus in the presence of CPT (Supplemental Figure 7A). Phosphorylation of TRIM28 on Ser824 by the ATM kinase is an important activation event in the DNA damage response (32). We observed that phosphorylation of TRIM28 on Ser824 was also strongly induced in the nucleus within 1 h of CPT treatment and correlated with the appearance of γH2ax staining for DNA damage by confocal microscopy (Figure 8B). Given the interaction between SRSF3 and TRIM28, we assessed colocalization of SRSF3 and TRIM28(pSer824) in HepG2 cells following CPT-induced DNA damage. SRSF3 was predominantly nuclear in both control and CPT-treated cells. SRSF3 and TRIM28(pSer824) showed nuclear colocalization in CPT-treated cells but did not colocalize in control cells in the absence of DNA damage (Figure 8C). A similar result was observed in colocalization of SRSF3 and TRIM28(pSer473) (Supplemental Figure 7B). Localization of SRSF3 and total TRIM28 did not correlate either with or without CPT-induced DNA damage in HepG2 cells (Figure 8D). SRSF3 was predominantly nuclear, but TRIM28 showed cytoplasm and perinuclear punctate staining (Figure 8D and Supplemental Figure 7C).

### A degradation-resistant SRSF3 (K11R) prevents HCC.

**Figure 9:**
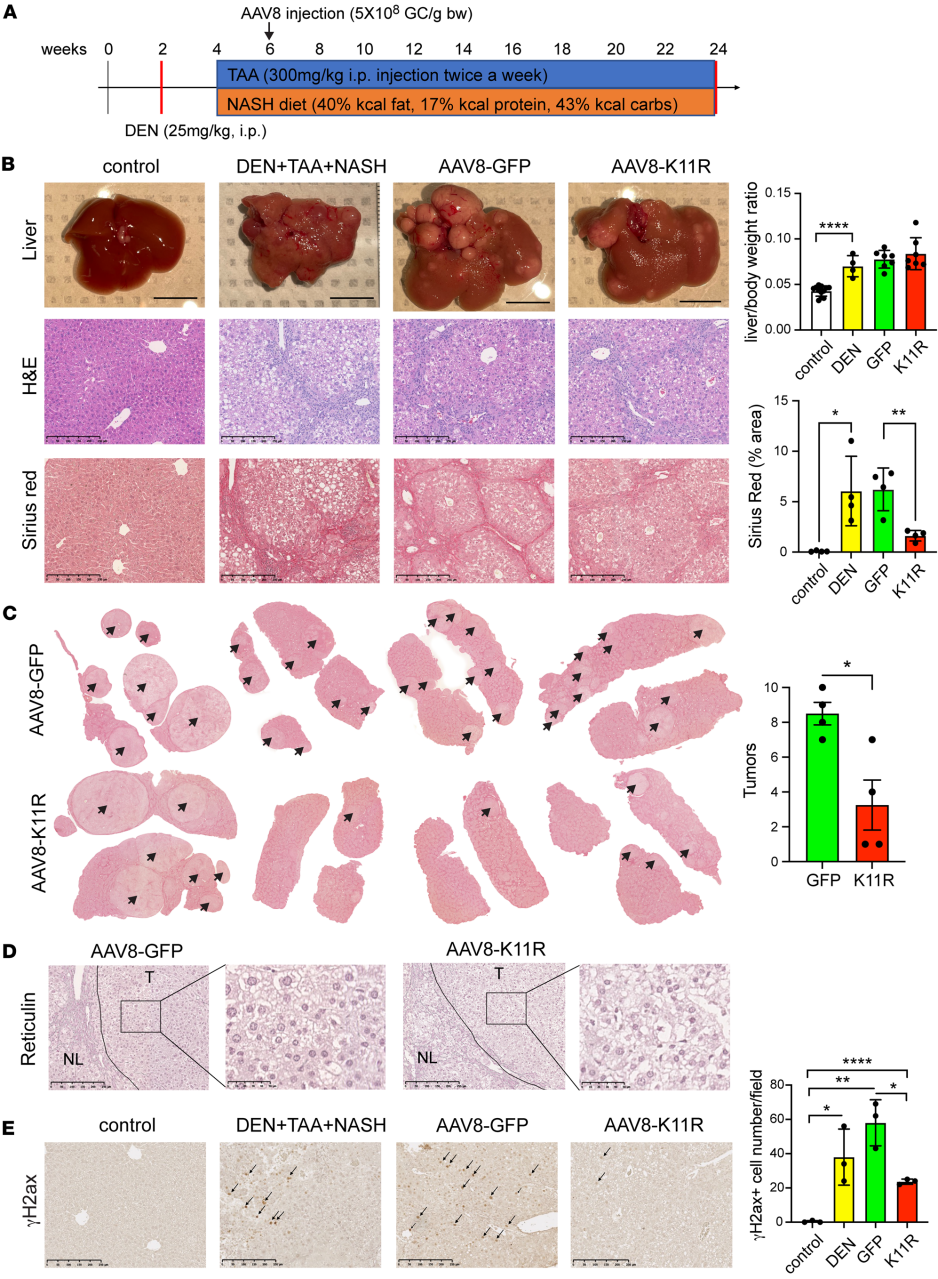
Preventing SRSF3 degradation reduces HCC. (**A**) Schematic of the chemically induced mouse HCC model. DEN (25 mpk, i.p.) was administered to mice at 2 weeks of age, mice were placed on Western diet starting at 4 weeks of age and injected with TAA (300 mpk, i.p.) twice a week for a further 20 weeks. AAV8 infection was performed by tail vein injection on week 6. (**B**) Whole liver images and H&E and Sirius red–stained sections from lean mice (control), HCC mice (DEN+TAA+NASH), HCC mice infected with AAV8-GFP (AAV8-GFP), and HCC mice infected with AAV8-SRSF3-K11R (AAV8-K11R). Scale bar for liver pictures: 1 cm. Scale bar for sections: 250 μm. Graphs show liver to body weight ratio (*n* = 4–7/group) and quantification of fibrosis by Sirius red staining (*n* = 4/group). Control mice are shown in white, DEN+TAA+MASH-treated (DEN) in yellow, GFP-infected in green, and K11R-infected in red. (**C**) Sirius red–stained whole liver sections from mice infected with AAV8-GFP or AAV8-K11R (*n* = 4/group) showing pale steatotic tumors (arrows). Graph shows quantification of tumor number. (**D**) Representative reticulin staining of liver sections from AAV8-GFP– or AAV8-K11R–infected mice. T, tumor; NL, adjacent nontumor liver. Scale bar for reticulin-stained sections: 250 μm. Scale bar for magnified sections: 50 μm. (**E**) Immunohistochemical staining for γH2ax (brown) and quantification of positive nuclei/field (*n* = 3/group). Black arrows indicate representative positive cells. Scale bar: 250 μm. All quantified results are presented as mean ± SD; *P < 0.05, **P < 0.01, ****P < 0.0001 by 1-way ANOVA or 2-tailed *t* test.

SRSF3-K11R prevented DNA damage in vitro and in vivo, so we then tested whether SRSF3-K11R would prevent the development of HCC in the context of MASH/cirrhosis. We chose a chemically induced HCC model (diethyl-nitrosamine [DEN]/thioacetamide [TAA]/MASH) to mimic human MASH-driven HCC (41). C57BL/6J mice were treated with the carcinogen DEN at 2 weeks of age to cause hepatic DNA damage. After weaning at 4 weeks, the mice were placed on the Western diet and treated with TAA to induce liver fibrosis and cirrhosis. By 24 weeks of age, these mice developed all the clinical features of MASH and HCC. We used AAV8 expression of SRSF3-K11R following injection of the virus through the tail vein (Figure 9A). Our AAV8 vectors only express for few weeks following injection, so we expressed SRSF3-K11R by tail vein injection at 6 weeks of age to test the effect of SRSF3-K11R during early carcinogenesis. All DEN/TAA/MASH mice developed tumors accompanied by elevated liver/body weight (Figure 9B). The H&E-stained and Sirius red–stained liver sections showed the expected hepatic steatosis and cirrhosis that was reduced by SRSF3-K11R (Figure 9B). Examination of Sirius red–stained liver sections showed reduced number of tumors in mice expressing SRSF3-K11R compared with GFP controls (Figure 9C). The identity of these tumors as HCC was confirmed by the absence of reticulin staining in the tumors but not the adjacent liver in both GFP– and SRSF3-K11R–expressing livers (Figure 9D). Liver sections from SRSF3-K11R mice also showed less DNA damage by γH2ax staining (Figure 9E). Thus, overexpression of SRSF3-K11R inhibited DNA damage and mitigated HCC growth.

## Discussion

R-loops and DNA damage have been associated with the development of HCC (42–45), and there is growing evidence that splicing factors and other RNA-binding proteins can influence R-loop–associated DNA damage and genome instability (14, 15, 21–23, 46). For example, depletion of the RNA splicing factor ASF1/SF2, also known as SRSF1, results in DNA double-strand breaks in a locus prone to R-loop formation (21, 22). Similarly depletion of the splicing factor XAB2 (15) or knockdown of Slu7 in cultured cells and mice livers causes R-loop accumulation and DNA damage (23). Interestingly, depletion of Slu7 causes expression of a truncated, dominant-negative form of SRSF3 that may cause R-loops and regulate splicing of the sister chromatid cohesion protein *Soronin* (23). Mutations in other splicing factors, including *SRSF2* and *U2AF1*, cause cell growth defects also through elevated R-loops (14).

How R-loops are linked to DNA damage is unclear. The lncRNA TUG1 has been implicated in R-loop resolution through interaction with replication protein A (RPA) bound to the single-stranded DNA loop and recruitment of the helicase DHX9 (47); XAB2 interacts with the DNA damage response genes ERCC1 and XPF/G for R-loop processing (15); the mitotic proteins BUB3 and BuGZ interact with the splicing machinery to suppress R-loops (48); and the DDX39/THO complex can prevent R-loops via association with YTHDC1 and m6A methylated RNA (49). In addition to these mechanisms, we found that SRSF3 interacted with components of the ATM DNA damage complex that are crucial in DNA repair and R-loop resolution (50, 51). Integrity of this SRSF3-TRIM28-MDC1 complex seemed to be dependent on expression of all proteins as knockdown of either SRSF3, TRIM28, or MDC1 affected the expression of the other proteins. The protein levels of TRIM28 and MDC1 were also decreased in livers of mice with MAFLD or MASH or following lipid overload in cells, both of which caused SRSF3 loss as well as DNA damage. These results suggest that loss of the essential DNA repair proteins TRIM28 and MDC1 might be the underlying cause of DNA damage induced by SRSF3 deletion.

MDC1 is recruited to break sites and acts as a scaffold protein to recruit other repair proteins to sites of DNA damage (33, 52–54). When a DSB is formed, MDC1 binds γ-H2AX via its BRCT domain and recruits phosphorylated ATM(pSer1981) to repair the DNA damage (55, 56). Thus, MDC1 loss could directly interfere with the DNA repair process. Interactions of SRSF3 with TRIM 28 and SPECC1L have also been reported in a large-scale proteomic analysis of breast cancer and HEK293 cells (57, 58). TRIM28 phosphorylation is an early event in the DNA damage response mediated by ATM kinase (59). Phosphorylation of TRIM28 on Ser824 is observed exclusively at sites of DNA damage and leads to chromatin relaxation, allowing access of the repair proteins to the damaged DNA (60). In contrast, phosphorylation of TRIM28 on Ser473 attenuates its binding to HP1 family proteins and reduces expression of proapoptotic genes (35). Although TRIM28-KO mice are prone to spontaneous liver cancer (38–40), TRIM28 is overexpressed in HCC, promotes proliferation, and predicts an unfavorable prognosis (61–63). In this regard, TRIM28 functions as both a tumor suppressor and an oncogene, as has been previously reported for SRSF3 (9, 64).

Although preventing SRSF3 degradation improves MASH (10), it is not clear whether this is related to R-loop suppression. Indeed, the only study linking R-loops to liver function reported that deleting *Rnaseh1* in hepatocytes inhibits R-loop clearance and also causes mitochondrial damage, impairs liver function, and causes degeneration and fibrosis (65). Other R-loop–interacting proteins — including DDX39, BUB3, and ZNF207 — have been implicated in HCC (48, 66–68), but their role in MASH is unknown. Interestingly, deficiency of the RNA-helicase DDX39 inhibits lipogenesis by decreasing the nuclear translocation and activation of SREBP1, so it could have a role in hepatic steatosis; furthermore, DDX39 is a target for TRIM28 ubiquitination (69, 70). The paucity of studies of R-loops in fatty liver disease highlights the need for further studies of both R-loops and their interacting proteins and whether they could play a causative role in disease progression.

In summary, our work indicates that stabilization of SRSF3 was important in maintaining genomic integrity. Loss of SRSF3 was observed during DNA damage and depletion of SRSF3 caused DNA damage via R-loop accumulation and loss of TRIM28 and MDC1, suggesting a feed-forward network driving DNA damage. The observation that inhibition of SRSF3 degradation prevents DNA damage in fatty liver disease may lead to more effective targeting therapeutic approaches for progressive liver disease as well as HCC.

## Methods

Supplemental Methods are available online with this article.

### Sex as a biological variable.

Our study included both male and female animals, and analysis did not discriminate based on sex. In MAFLD and MASH models, C57BL/6J mice were placed on high-fat diet (60% fat) for 12 weeks to induce MAFLD or Western Diet (40% fat, 0.2% cholesterol) to induce MASH. AAV8-GFP, AAV8-SRSF3-WT, and AAV8-SRSF3-K11R Flag-tagged vectors were injected through the tail vein at an inoculum of 1 × 10^10^ pfu AAV8 per mouse (10). The infected mice were placed on Western diet for 7 weeks then sacrificed for further analysis.

In the chemically induced HCC model, C57BL/6J mice were injected with diethylnitrosamine (25 mg/kg, i.p.) at 2weeks of age. After weaning, the mice were placed on Western diet and received TAA (300 mg/kg, i.p., 2× week) to induce liver injury and fibrosis.

In TRIM28 hepatocyte-specific KO mice, heterozygous *Trim28*^fl/+^ *Alb-Cre*^+^ mice were bred with *Trim28^fl/fl^* mice to obtain homozygous *Trim28^fl/fl^ Alb-Cre*^+^ animals (TRIM28-HKO) and their control cre-negative littermates (Flox).

### Cell culture, treatment and transfection.

Primary human hepatocytes were obtained from the Human Hepatocyte Isolation Distribution (HHID) program (University of Pittsburgh, Pittsburgh, Pennsylvania, USA) and were cultured in William’s Medium E supplemented with 10% FBS and 1% GlutaMax. For in vitro knockdown experiments, siRNAs (20 nM) were transfected into HEK293 cells or human hepatocytes using Lipofectamine RNAiMAX Transfection Reagent and into HepG2 cells via electroporation following the manufacturers’ protocols. After 48 hours, the cells were harvested for further analysis. Following SRSF3 knockdown in HEK293 cells, RNase H was transfected using Pierce Protein Transfection Reagent into HEK293 cells. Five units of RNase H were used for each well for 4 hours in 24-well plates.

### R-loop assays.

For the dot blot, genomic DNA samples were diluted to 50 ng/μL and 2 μL of each sample were spotted onto nitrocellulose membrane, crosslinked with UV light, blocked in 5% BSA for 1 hour at room temperature, and incubated with anti–DNA-RNA Hybrid antibody (S9.6, Kerafast, catalog EMH001) or anti–double-stranded DNA antibody (HYB331-01, Santa Cruz, catalog sc-58749) overnight at 4°C. To control for nonspecific binding by antibody S9.6, the nucleic acid samples were also digested with 5 units RNaseH for 30 minutes at 37°C to digest R-loops before dot blot analysis. R-loop imaging was performed in HEK293 cells following Srsf3 knockdown using antibody S9.6. Control samples were preincubated with 5 units RNaseH prior to the primary antibody. R-loop profiling was performed using a tagmentation approach (Active Motif) and analyzed using the HOMER software suite (71) and visualized using IGV (72).

### Other.

Western blots, coimmunoprecipitations, histochemical, and immunofluorescence staining were performed according to standard protocols (10).

### Statistics.

Data are presented as mean ± SD of at least 3 independent experiments. For normally distributed data, statistical analysis was performed using 1-way ANOVA or 2-tailed Student’s *t* tests unless mentioned otherwise. For nonnormally distributed data, statistical analysis was performed using a Mann-Whitney *U* or Kolmogorov-Smirnov nonparametric test as appropriate. All statistical analysis was performed using Prism v.8.0 (GraphPad). A statistically significant difference was defined as *P* < 0.05.

### Study approval.

All animal work was performed according to ARRIVE guidelines and was approved by the University of California San Diego IACUC.

### Data availability.

The data that support the findings of this study are available in the main text or the supplemental materials; values for all data points in graphs are reported in the Supporting Data Values file. Sequence data are available in SRA (https://www.ncbi.nlm.nih.gov/sra/?term=PRJNA1314818).

## Author contributions

Conceptualization was contributed by PW and NJGW; methodology was contributed by PW, MD, and LJJ; formal analysis was contributed by PW, LJJ, and NJGW; investigation was contributed by PW, MD, DK, Y Wang, Y Wu, and YJ; writing of the original draft was contributed by PW; review and editing were contributed by NJGW; supervision was contributed by NJGW; project administration was contributed by NJGW; and funding acquisition was contributed by NJGW. All authors have read and agreed to the published version of the manuscript.

## Funding support

This work is the result of NIH funding, in whole or in part, and is subject to the NIH Public Access Policy. Through acceptance of this federal funding, the NIH has been given a right to make the work publicly available in PubMed Central.

- VA Merit Review Award (I01BX004848) to NJGW.
- Senior Research Career Scientist Award (IBX005224) to NJGW.
- NIH grant (R01CA196853).
- Moores Cancer Center grant (P30CA023100).
- UCSD/UCLA Diabetes Research Center grant (P30DK063491).
- San Diego Digestive Disease Research Center grant (P30DK120515).

## Supplementary Material

Supplemental data

Unedited blot and gel images

Supplemental tables 1-2

Supporting data values

## Version 1 01/09/2026

Electronic publication
