# Supplementary material for: SRSF3-TRIM28-MDC1 prevents DNA damage caused by R-loops in fatty liver disease in mice
Source: JCI Insight. 2026 Jan 9;11(1):e188629. doi: 10.1172/jci.insight.188629 (PMC12890501; doi:10.1172/jci.insight.188629)
Supplement: Supplemental data [file jciinsight-11-188629-s017.pdf]

Supplementary Materials and Methods

Animal studies. Sex as a Biological Variable: both male and female mice were used for the studies. MAFLD and MASH models: C57BL/6J mice were purchased from Jackson Laboratories (Bar Harbor, ME) and housed in a 12-hour light/12-hour dark cycle. 8-week-old WT mice were placed on high-fat diet (HFD; 60% fat, D12492, Research Diets Inc., New Brunswick, NJ) to induce MAFLD or Western Diet (40% fat, 0.2% cholesterol, D12079B, Research Diets Inc.) to induce MASH for 12 weeks. Adeno-associated virus expression of degradation-resistant SRSF3 for MASH prevention: AAV8-GFP, AAV8-SRSF3-WT, and AAV8-SRSF3-K11R Flag-tagged vectors were injected into male C57BL/6J WT mice by the tail vein at an inoculum of $1x10^{10}$ pfu AAV8 per mouse. The AAV8 vectors were produced by the GT3 facility core (Gene Transfer, Targeting and Therapeutics Viral Vector Core) of the Salk Institute for Biological Studies (La Jolla, CA). The infected mice were placed on Western Diet (40% fat, 0.2% cholesterol) for 7 weeks then sacrificed for further analysis.

Chemically-induced HCC model: C57BL/6J mice were injected with diethylnitrosamine (DEN; 25 mg/kg, i.p.; N0756, Sigma-Aldrich, St. Louis, MO) at 2-weeks of age. After weaning at 4 weeks, the mice were placed on Western diet (40% fat, 0.2% cholesterol) and received thioacetamide (TAA); 300 mg/kg, i.p., 2x week) to induce liver injury and fibrosis. These mice were injected with AAV8-GFP and AAV8-SRSF3-K11R viral vectors through the tail vein with $5x10^{8}$ pfu/g body weight. Mice were euthanized at 24 weeks for the assessment of tumors.

SRSF3 hepatocyte-specific knockout mice: Srsf3tm1Pjln mice (*Srsf3flox:flox*) mice contain a floxed *Srsf3* allele with LoxP sites flanking exons 2 and 3 of *Srsf3*. Floxed *Srsf3* mice were mated to *TgN*(*Alb-Cre*)*21Mgn* transgenic mice that express cre recombinase in hepatocytes driven by the rat albumin enhancer/promoter. The resulting heterozygous *Srsf3flox*/+;*Alb-Cre* + mice were bred with *Srsf3flox/flox* mice to obtain homozygous *Srsf3flox/flox;Alb-Cre* + animals (SRSF3-HKO) and their control cre-negative littermates. Similarly, we generated *Srsf3flox/flox;Alb-Cre^{ERTM}* + (SRSF3-conditional KO) with a tamoxifen-inducible CRE expressed from the *Alb* promoter to allow acute deletion of *Srsf3* in mature hepatocytes.

TRIM28 hepatocyte-specific knockout mice: B6.129S2(SJL)-Trim28tm1.1Ipc/J mice (*Trim28flox:flox*) were a generous gift from Dr. Hyun Kyoung Lee (Department of Pediatrics-Neurology, Jan and Dan Duncan Neurological Research Institute, Baylor College of Medicine and Texas Children's Hospital, Houston, TX). *Trim28flox:flox* mice contain two LoxP sites introduced into intron 3 and intron 14. Heterozygous *Trim28flox*/+;*Alb-Cre* + mice were obtained by mating floxed *Trim28* mice to *TgN*(*Alb-Cre*)*21Mgn* mice. The resulting heterozygous *Trim28flox*/+;*Alb-Cre* + mice were bred with *Trim28flox/flox* mice to obtain homozygous *Trim28flox/flox;Alb-Cre* + animals (TRIM28-HKO) and their control cre-negative littermates (Flox).

Cell culture, treatment and transfection. HepG2 cells were obtained from ATCC (Manassas, VA) and maintained in 1x MEM (15-010-CV, Corning, Glendale, AZ) supplemented with 10% fetal bovine serum (F4135, Sigma-Aldrich) and 1% GlutaMax supplement (35050061, Gibco, Thermo-Fisher, Waltham, MA) at 37°C in an atmosphere of 5% CO_2. HEK293 cells were obtained from ATCC and maintained in 1x DMEM (10-014-CV, Corning) supplemented with 10% fetal bovine serum at 37°C in an atmosphere of 5% CO_2. Primary human hepatocytes were obtained from the Human Hepatocyte Isolation Distribution (HHID) program (Univ. Pittsburgh, Pittsburgh, PA) and were cultured in William's Medium E (A1217601, Gibco) supplemented with 10% FBS and 1% GlutaMax at 37°C in an atmosphere of 5% CO_2.

Palmitic acid (PA; P0500, Sigma-Aldrich), stearic acid (SA; L1012, Sigma-Aldrich) and linoleic acid (LA; S4751, Sigma-Aldrich) were solubilized in 100 mM methyl-β-cyclodextrin (MBCD; C4555, Sigma-Aldrich) and incubated with the HepG2 cells at a final concentration of 500 μM for 12 h. Then the cells were collected for further analysis or experiment. Camptothecin (CPT; C9911, Sigma-Aldrich) was dissolved in DMSO. ATM inhibitor (KU-55933) (3544, Tocris) was dissolved in DMSO and incubated with the HepG2 cells at a final concentration of 5 μM for 1h before CPT treatment. NEDD8-activating enzyme (NAE) inhibitor (MLN4924) (A11260, AdooQ Bioscience) was dissolved in DMSO and incubated with the HepG2 cells at a final concentration of 3 μM for 1h before CPT treatment.

For the DNA damage prevention study, HepG2 cells were infected with AAV8-GFP, AAV8-SRSF3-WT or AAV8-SRSF3-K11R at an m.o.i. of 500,000 for 48 h. The cells were then treated with 0.1 μM CPT for 1 h, 200 μM hydrogen peroxide (H_2O_2) for 1h, 500 μM PA for 12 h and processed for assessment of DNA damage. For assessment of DNA damage in HEK293 cells, cells were transfected in 6-well plates with 1 μg GFP, Flagged-SRSF3-WT, or Flagged-SRSF3-K11R expression plasmids using EndoFectin™ Max transfection reagent (EF013, GeneCopoeia, Rockville, MD) following the manufacturer's protocol. The cells were treated with 0.1 μM CPT for 1 h and genomic DNA extracted for R-loop detection.

For in vitro knockdown experiments, siRNAs (20 nM) were transfected into HEK293 cells or human hepatocytes using Lipofectamine RNAiMAX Transfection Reagent (13778100, ThermoFisher), and into HepG2 cells via electroporation (Neon Transfection System, MPK1096, ThermoFisher) following the manufacturers' protocols. After 48 h, the cells were harvested for further analysis. Following SRSF3 knockdown in HEK293 cells, RNase H (NEB #M0297) protein was transfected using Pierce Protein Transfection Reagent (89850, ThermoFisher) into HEK293 cells. Five units of RNase H were used for each well for 4 h in 24-well plate.

Western blot. Cells or tissues were harvested and lysed with RIPA lysis buffer (150 mM NaCl, 50 mM Tris•HCl pH 7.4, 1% NP-40, 0.5% sodium deoxycholate, 0.1% SDS) on ice for 2 h. Protease and phosphatase inhibitor cocktail (78441, ThermoFisher) was freshly added in RIPA buffer each time before use. The lysates were

sonicated and clarified by centrifugation at 13000 x g for 15 min (4 °C) and the supernatants were collected for immunoblot. Equal amounts of cellular protein (10 µg) were separated by SDS/PAGE using 10-12% gels, transferred to PVDF membranes (MilliporeSigma), blocked with 5% BSA for 1 h at room temperature, and immunoblotted with primary antibodies overnight at 4 °C followed by HRP-conjugated secondary antibodies at room temperature for 1 h. Membranes were washed 3 times in 0.1%TBST, and then developed using a chemiluminescent substrate kit (34577, ThermoFisher). Antibodies used for immunoblotting were mouse monoclonal 7B4 anti-SRSF3 (1:1000 dilution, ATCC, CRL-2384), anti-γH2ax rabbit monoclonal (1:1000 dilution, 2577S, Cell Signaling Technology, Danvers, MA), anti-β-actin mouse monoclonal (1:2000 dilution, sc-47778, Santa Cruz Biotechnology, Santa Cruz, CA), anti-TRIM28 rabbit monoclonal (1:1000 dilution, 13435S, Cell Signaling Technology), anti-TRIM28 phosphoSer473 mouse monoclonal (1:1000, 654101, BioLegend, San Diego, CA), anti-MDC1 mouse monoclonal (1:1000 dilution, M2444, Sigma-Aldrich), anti-BRCA1 mouse monoclonal (1:1000 dilution, MA1-23164, Invitrogen, ThermoFisher), anti-53BP1 rabbit polyclonal (1:1000 dilution, PA1-16565, Invitrogen), anti-Flag mouse monoclonal (1:1000 dilution, F3165, Sigma-Aldrich), anti-HA rabbit polyclonal (1:1000 dilution, sc-805, Santa Cruz), anti-FLAG mouse monoclonal (1:1000 dilution, F7425, MilliporeSigma), HRP-conjugated anti-mouse secondary antibody (1:4000 dilution, sc-516102, Santa Cruz Biotechnology), and HRP-conjugated anti-rabbit secondary antibody (1:4000 dilution, sc-2357, Santa Cruz Biotechnology).

Co-immunoprecipitation (Co-IP). The Flag-SRSF plasmids were obtained from Takbum Ohn (Chosun University, Gwangju, Republic of Korea). Primary hepatocytes, or HEK293 cells co-transfected with Flag-tagged SRSF3-WT, Flag-tagged SRSF3-K11R, and HA-tagged TRIM28, were collected, washed once in PBS and lysed in RIPA lysis buffer with freshly added protease and phosphatase inhibitor cocktail (Thermofisher) for 2 h at 4 °C. The lysates were briefly sonicated and clarified by centrifugation at 13000 x g for 15 min (4 °C). Protein G Beads (Dynabeads, ThermoFisher) were pre-incubated with primary antibodies for 10 min at room temperature followed by incubation at 4 °C overnight with rotation. The primary antibodies used were an anti-FLAG rabbit mAb (F7425, MilliporeSigma), or an anti-SRSF3 mouse mAb (7B4, ATCC) antibody. The lysate supernatants (~700 mg protein) were incubated with primary antibody-conjugated beads overnight at 4°C with rotation. Beads were washed and bound proteins were eluted by heating at 70 °C for 10 min in SDS-sample buffer (Bio-Rad). The eluates were analyzed by SDS-PAGE and immunoblotting as above.

Immunofluorescence (IF). The cells were grown, treated, fixed and stained directly in multi-well plates or on coverslips. The cells were covered with 4% formalin in 1X PBS for 15 min at room temperature, rinsed three times in 1x PBS and permeabilized with 0.5% Triton X-100 for 20 min. Samples were blocked in 5% normal goat

serum in PBS, 1% Triton X-100 for 1 h, then incubated with anti-γH2ax rabbit antibody (1:400 dilution, 2577S, Cell Signaling Technology), or anti-γH2ax mouse antibody (1:400 dilution, 14-965-80, Invitrogen) diluted in PBS, 0.1% Triton X-100 (1:400 dilution) overnight at 4 °C with gentle rocking. After the cells were washed three times in PBS for 10 min each, they were incubated with 1:800 dilution of Alexa Fluor 488-conjugated anti-rabbit IgG and 1:800 dilution (A11008, Invitrogen) of Alexa Fluor 555-conjugated anti-mouse IgG (A21422, Invitrogen) at room temperature in dark. After PBS wash, the cells were incubated with 300 nM DAPI stain solution at room temperature for 5 min in the dark. Staining was visualized by confocal laser-scanning microscopy (Leica SP8 with lightning deconvolution) or Keyence Fluorescent Microscope after mounting.

For R-loop imaging, HEK293 cells were fixed with cold methanol and washed three times with PBS. After fixation, cells were treated with RNase H (10 U/ml; NEB #M0297) at 37 °C for 1 hour, followed by three PBS washes. Cells were then blocked with 5% BSA in TBS for 1 hour. The S9.6 antibody was diluted 1:200 in 5% BSA/TBS and incubated overnight at 4 °C. AF555-conjugated mouse secondary antibody was diluted 1:200 and incubated for 1 hour at room temperature. Nuclei were counterstained with DAPI (1:500), and images were acquired using the UCSD Microscopy Core (NINDS NS047101) Leica SP8 confocal microscope. Immunofluorescence signals were quantified using ImageJ.

Immunohistochemistry (IHC). IHC was performed on formalin-fixed, paraffin-embedded (FFPE) mouse liver sections. Tissues were deparaffinized rehydrated in xylene and rehydrated in series of graded alcohols. Then the slides were placed in a vessel full of sodium citrate buffer and steamed for 20 min for antigen retrieval. After the slides were cooled down in room temperature and rinsed twice in water, they were immersed in 0.6% H_2O_2 for 10 min to inactivate endogenous peroxidase. After rinsed in PBST, the slides were blocked with 5% normal goat serum in PBST for 1 h then incubated with anti-γH2ax antibody (1:400 dilution, 2577S, Cell Signaling Technology) overnight at 4°C with gentle rocking. The slides were incubated with a biotinylated secondary antibody (VECTASTAIN® ABC-HRP Kit, Rabbit, PK-4001, Vector Labs, Newark, CA) at room temperature for 1 h followed by avidin peroxidase (VECTASTAIN® ABC-HRP Kit, Rabbit, PK-4001, Vector Labs). After PBS wash, the slides were visualized by substrate mixture (DAB Substrate Kit, SK-4105, Vector Labs), counterstained with hematoxylin and mounted.

Genomic DNA purification and R-loop dot blot. Liver tissue samples (10 mg) were homogenized in TRIzol reagent (15596026, Invitrogen) (1 ml/50-100 mg) by VWR 200 homogenizer. Cells were dissociated by trypsin (25200056, Gibco) and washed by PBS. Following centrifugation at 300 rcf for 5 min at 4 °C, the cell pellet was lysed in cold cell lysis buffer (80 mM KCl, 5 mM PIPES pH 8.0, 0.5% NP-40) and incubated on ice for 10 min. After centrifugation at 500 x g for 5 min, the nuclear pellet was lysed in TRIzol reagent. Following

homogenization, genomic DNA was isolated following the manufacturer's protocol. Briefly, samples were lysed and chloroform was used to separate phases. Pure ethanol was added to the interphase and the lower phenol-chloroform phase to pellet DNA. The DNA pellet was washed in 0.1 M sodium citrate in 10% ethanol 2-3 times followed by 75% ethanol wash once. Then the DNA pellet was dissolved in 8 mM NaOH and adjusted to pH 7-8 with 1M HEPES. The DNA samples were diluted to 50 ng/µL and 2 µl of each sample were spotted onto nitrocellulose membrane (WHA10401316, Millipore) then crosslinked with UV light (1200 µJ x 100, Stratalinker UV crosslinker 1800, Stratagene, La Jolla, CA). The membrane was blocked in 5% BSA for 1 h at room temperature then incubated with anti-DNA-RNA Hybrid [S9.6] Antibody (1:1000, ENH001, Kerafast, Boston, MA) or anti-ds DNA Marker Antibody (1:2000, sc-58749, Santa Cruz) overnight at 4 °C, followed by TBST wash and HRP conjugated secondary antibody (anti-mouse, 1:4000 dilution, sc-516102, Santa Cruz) incubation. The membrane was washed in TBST 3 times and then developed using a chemiluminescent substrate kit (34577, ThermoFisher). To control for non-specific binding by antibody S9.6, the nucleic acid samples were also digested with 5 units RNase H (#M0297, New England BioLabs, Ipswich, MA) for 30 min at 37 °C to digest R-loops before dot-blot analysis.

R-loop profiling. Primary hepatocytes were prepared from *Srsf3flox/flox;Alb-Cre^{ERTM}* + mice to allow acute deletion of *Srsf3*. Deletion was initiated by treating cells in triplicate with tamoxifen or vehicle for 48 h then cells were harvested for R-loop profiling using a tagmentation approach (CUT&Tag-IT R-loop Assay Kit #53167, Active Motif, Carlsbad, CA). Hepatocytes were processed according to manufacturer's protocol and libraries prepared using Tn5 transposase. Amplified DNA was quantified on an Agilent tape station sequenced on Illumina NovaSeq (PE150). Raw reads were trimmed for primers and short, unpaired reads filtered out before aligning to the mm10 genome using STAR. Tag directories were created using the HOMER suite of ChIPseq tools (*Mol Cell.* 2010;38(4):576-89; Benner Lab, UCSD), R-loop peaks identified using findPeaks and differential peaks identified using getDifferentialPeaksReplicates.pl. Peaks were annotated using annotatePeaks.pl and motifs found using findMotifsGenome.pl. BedGraph files of R-loop peaks and SRSF3 eCLIP peaks were visualized using the Integrated Genome Viewer (*Nat Biotechnol.* 2011;29(1):24-6; Mesirov Lab, UCSD).

Immunoprecipitation-Mass Spectrometry. Mouse primary hepatocytes were obtained by 2-step perfusion with liver perfusion medium SC-1 (137 mM NaCl, 5.4 mM KCl, 0.56 mM NaH_2PO_4•H_2O, 0.85 mM Na_2HPO_4, 10 mM HEPES, 4.2 mM $NaHCO_3$, 0.5 mM EGTA, and 5 mM glucose), followed by digestion medium SC-2 (137 mM NaCl, 5.4 mM KCl, 0.56 mM NaH_2PO_4•H2O, 0.85 mM Na_2HPO_4, 10 mM HEPES, 4.2 mM $NaHCO_3$, and 12 mM $CaCl_2$•H_2O) containing 0.5 mg/mL collagenase D (Roche, Basel, Switzerland). The hepatocytes were lysed and incubated with anti-SRSF3 antibody conjugated beads. After elution and protein digestion, the peptides

were analyzed by LC-MS/MS at the Proteomics Core at the Sanford Burnham Prebys Medical Discovery Institute (La Jolla, CA).

Statistics. Values in bar and line graphs are presented as mean ± SD of at least three independent experiments for in vitro experiments, or a minimum of three mice for in vivo experiments. For normally distributed data, statistical analysis was performed using 1-way ANOVA or two-tailed Student's t-tests unless mentioned otherwise. For non-normally distributed data, statistical analysis was performed using Mann-Whitney or Kolgomorov-Smirnov non-parametric test as appropriate. All statistical analysis was performed using Prism v.8.0 (GraphPad, La Jolla, CA). A statistically significant difference was defined as *$P<0.05$, **$P<0.01$, ***$P<0.001$, ****$P<0.0001$.

Study Approval: All animal work was reviewed and approved by the University of California San Diego Institutional Animal Care and Use Committee.

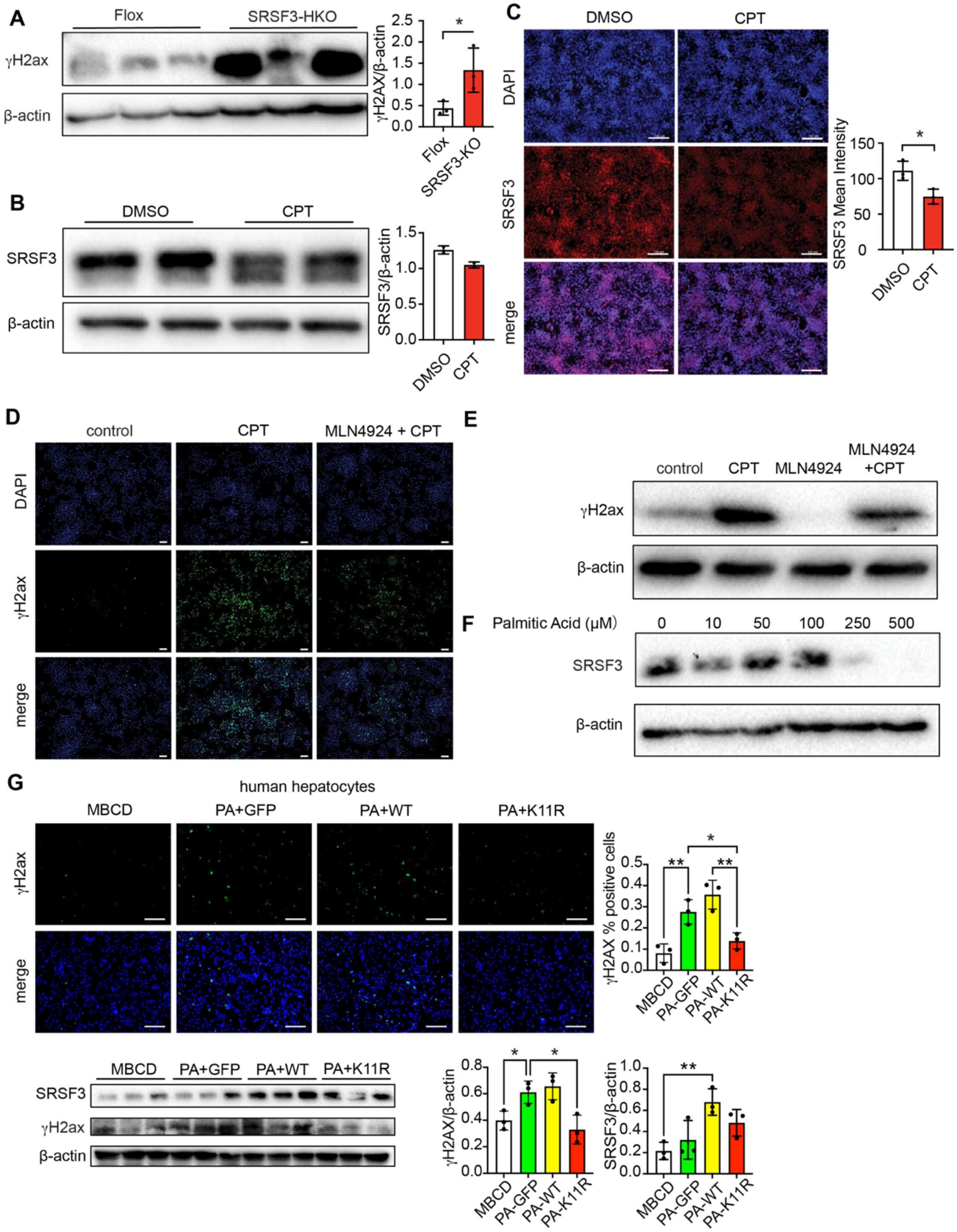

Figure S1: SRSF3 loss accompanied with DNA damage. Immunoblots of γH2ax in hepatocytes of Flox and SRSF3-HKO mice. Graph shows quantification of γH2ax protein levels normalized to β-actin (*n* = 3/group). (**B&C**) Immunoblotting and immunofluorescence staining of SRSF3 of HepG2 cells treated with DMSO or CPT (5 μM) for 1 h. Scale bars: 100 μm. Graph shows quantification of SRSF3 protein levels normalized to β-actin (*n* = 2/group) and quantification of SRSF3 mean intensity (*n* = 3/group). **(D)** Immunofluorescence staining for γH2ax in HepG2 cells treated with or without the NAE-1 inhibitor (MLN4924, 3 μM) for 1 h. Then cells were treated with DMSO as control or CPT (5 μM) for 5 h. DAPI was used to visualize the nucleus. Scale bars: 50 μm. **(E)** Immunoblots of γH2ax in HepG2 cells treated with the NAE-1 inhibitor as above. **(F)** Immunoblots of SRSF3 in HepG2 cells treated with PA at different doses (0-500 μM). **(G)** Human primary hepatocytes were infected by AAV8 expressing GFP, SRSF3-WT, SRSF3-K11R directly at MOI 50,000 for 48h. γH2ax was detected by immunofluorescence staining (green) and immunoblot following treatment with 250 μM palmitic acid for 12 h. Scale bars: 100 μm. SRSF3 was detected by immunoblot. Graph shows quantification of SRSF3 and γH2ax protein levels normalized to β-actin (*n* = 3/group) and quantification of γH2ax-positive nuclei/field (*n* = 3/group). All quantified results are presented as mean ± SD; **P* < 0.05 by t-test.

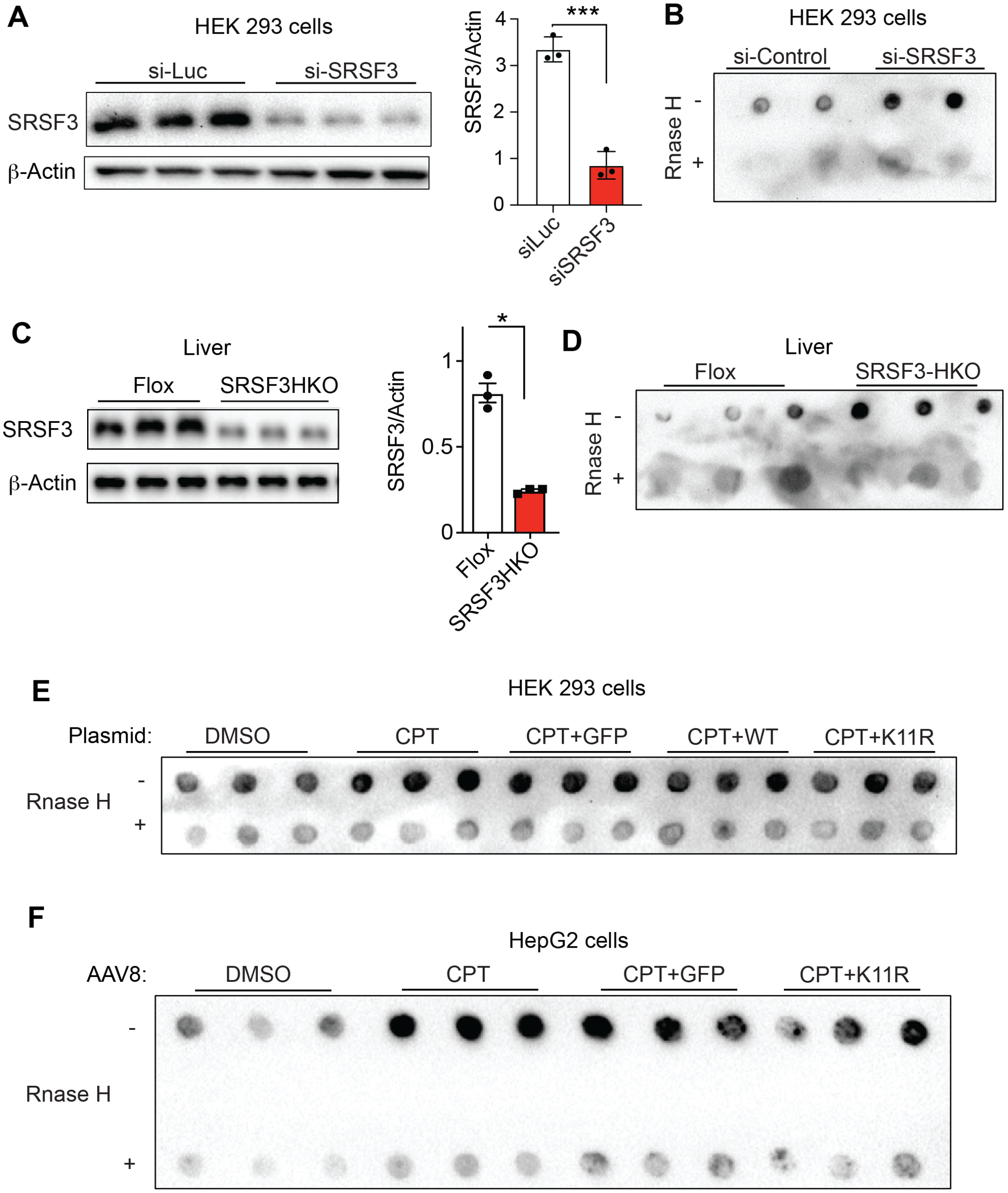

Figure S2: RNase H treatment reduces S9.6 recognition of R-loops. (**A**) Immunoblotting for SRSF3 in HEK293 cells with siRNA knockdown of Srsf3. **(B)** Dot-blot of R-loops using S9.6 antibody in genomic DNA from HEK293 cells treated with control siRNA or SRSF3 siRNA. Five Units of RNase H were added to parallel samples to digest RNA-DNA hybrids. (**C**) Immunoblotting for SRSF3 in liver extracts from control (Flox) or SRSF3 KO mice (SRSF3HKO). (**D**) Dot-blot of R-loops in genomic DNA from Flox hepatocytes and SRSF3 knockout hepatocytes with RNase H treatment to digest R-loops. (**E**) Dot-blot of R-loops in genomic DNA from HEK293 cells expressing GFP, Flagged-SRSF3-WT, Flagged-SRSF3-K11R and then treated with 0.1 µM CPT or DMSO for 1 h with RNase H treatment to digest R-loops. (**F**) Dot-blot of R-loops using S9.6 antibody in genomic DNA from HepG2 cells expressing GFP or Flagged-SRSF3-K11R and treated with CPT with RNase H treatment to digest R-loops.

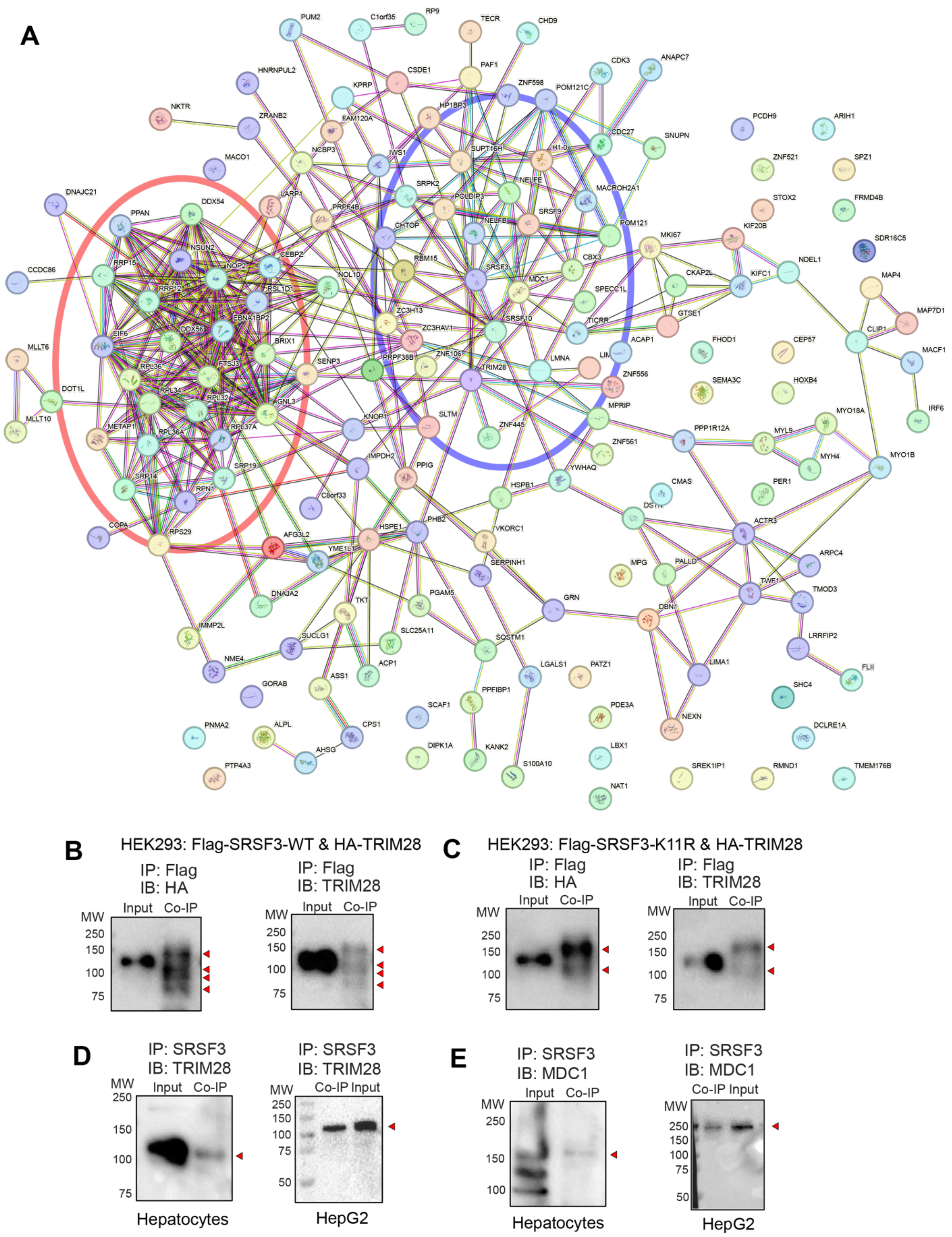

Figure S3: SRSF3 interacts with TRIM28 and MDC1. (**A**) String protein-protein interaction network from SRSF3-interacting proteins by LC-MS/MS. Proteins associated with ribosome are circled in red. SRSF3-interacting proteins are circled in blue. Single lines indicate new interactions from our proteomic data, multiple lines indicate previously known interactions from public databases. HEK 293 cells were co-transfected with HA-TRIM28 and Flagged-SRSF3-WT (**B**) or Flagged-SRSF3-K11R (**C**) plasmids for 48 h. Cell lysates were immunoprecipitated (IP) with anti-Flag antibody and then immunoblotted (IB) for HA and TRIM28. Input indicates input control, Co-IP indicates the co-precipitated proteins (red triangles). Mouse hepatocytes lysates were immunoprecipitated (IP) with antibody against SRSF3 and then immunoblotted (IB) for TRIM28 **(D)** or MDC1 **(E)**. Input indicates input control, Co-IP indicates the co-precipitated proteins (red triangles).

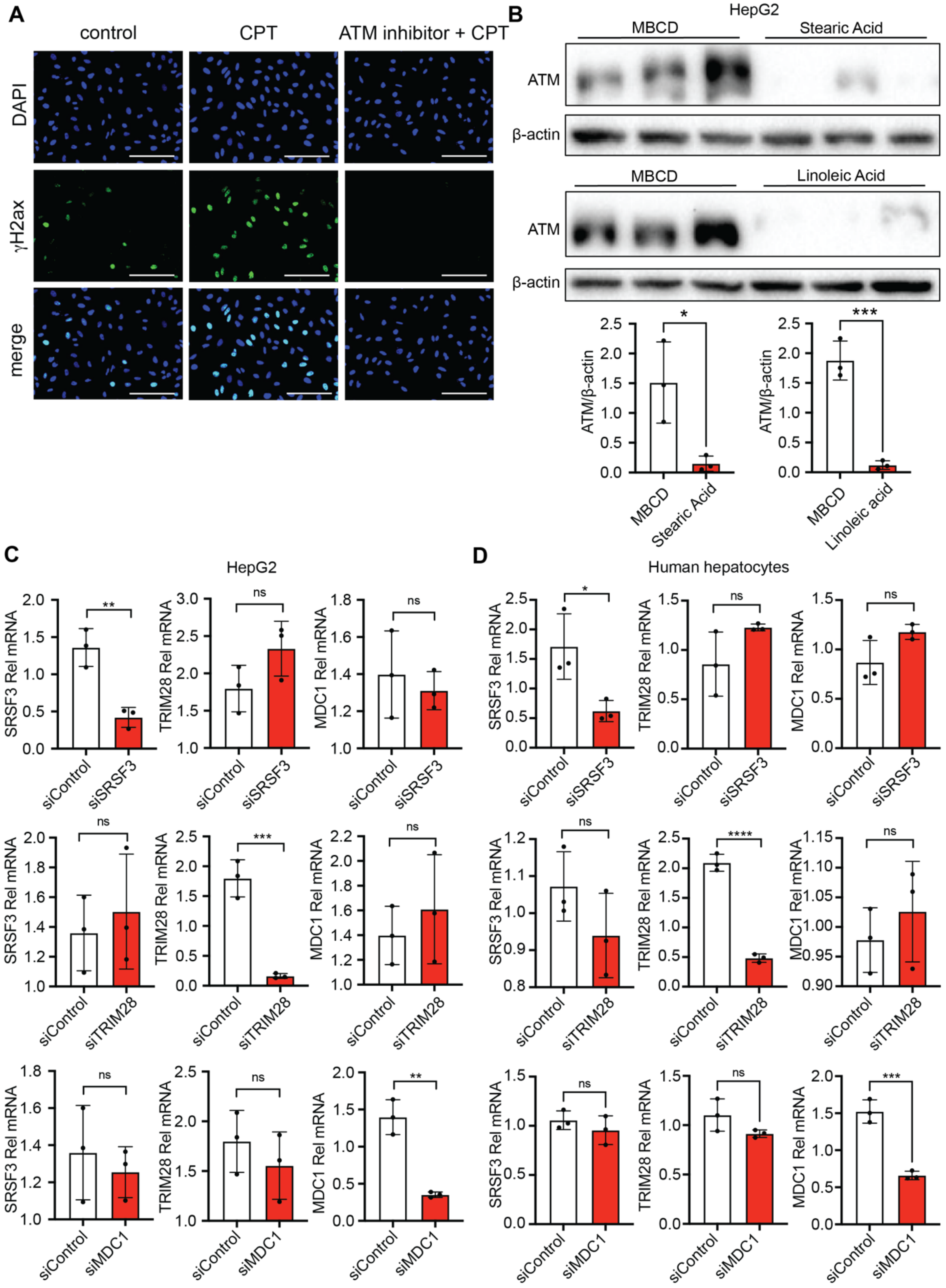

Figure S4: DNA damage is ATM dependent and *SRSF3, TRIM28* and *MDC1* mRNA levels are unchanged. (A) Immunofluorescence staining for γH2ax in HepG2 cells treated with CPT (5 μM) for 1 h in the absence or presence of an ATM kinase inhibitor (KU-55933, 5μM, 1h). DAPI was used to visualize the nuclei. **(B)** Immunoblots for ATM levels in HepG2 cells treated with stearic acid or linoleic acid (500 μM) complexed to MBCD for 12 h. All quantified results are presented as mean ± SD; *$P < 0.05$, ***$P < 0.0001$ by t-test. **(C-D)** Expression of SRSF3, TRIM28 and MDC1 genes by qPCR in HepG2 cells **(C)** or human primary hepatocytes **(D)** with SRSF3 knockdown, TRIM28 knockdown and MDC1 knockdown by siRNA (40 nM, 48 h). Expression is normalized to Peptidylprolyl isomerase A (Ppia) (n = 3/group). All quantified results are presented as mean ± SD; **P < 0.01, ***P < 0.001 by t-test.

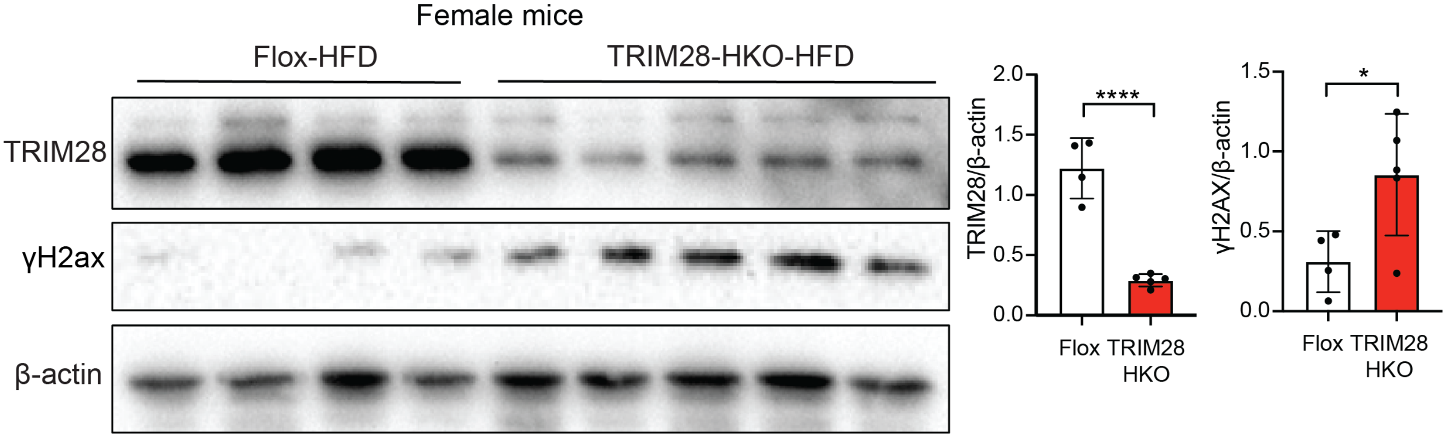

Figure S5: TRIM28 deletion causes DNA damage in female mice and TRIM28 and MDC1 are elevated in human HCC and inversely correlate with survival. Immunoblots of γH2ax and TRIM28 in hepatocytes from female Flox mice and TRIM28-HKO mice on high-fat diet (HFD) for 16 weeks. Graph shows quantification of protein levels normalized to β-actin (n = 4-5/group). Flox mice shown in white, TRIM28-HKO mice in red. All quantified results are presented as mean ± SD; **P < 0.01, ***P < 0.001 by t-test.

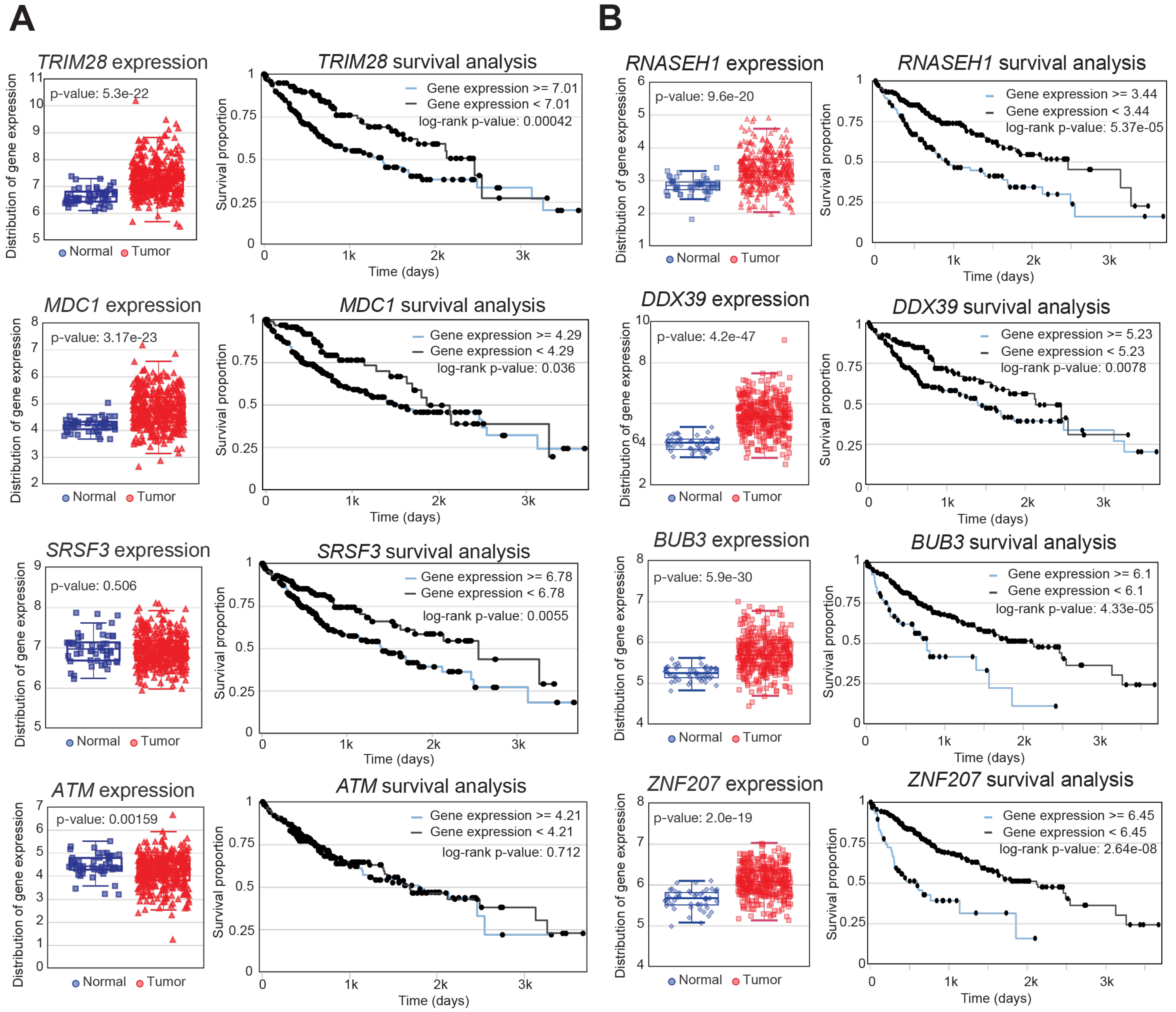

Figure S6: TRIM28 and MDC1 are elevated in human HCC and inversely correlate with survival. **(A)** Expression levels and survival curves for *TRIM28, MDC1, SRSF3* and *ATM* in HCC data from the TCGA. **(B)** Expression levels and survival curves for *RNASEH1, DDX39, BUB3* and *ZNF207* in HCC data from the TCGA. P-value indicates significance for expression plots and log-rank p-value indicates significance for survival plots.

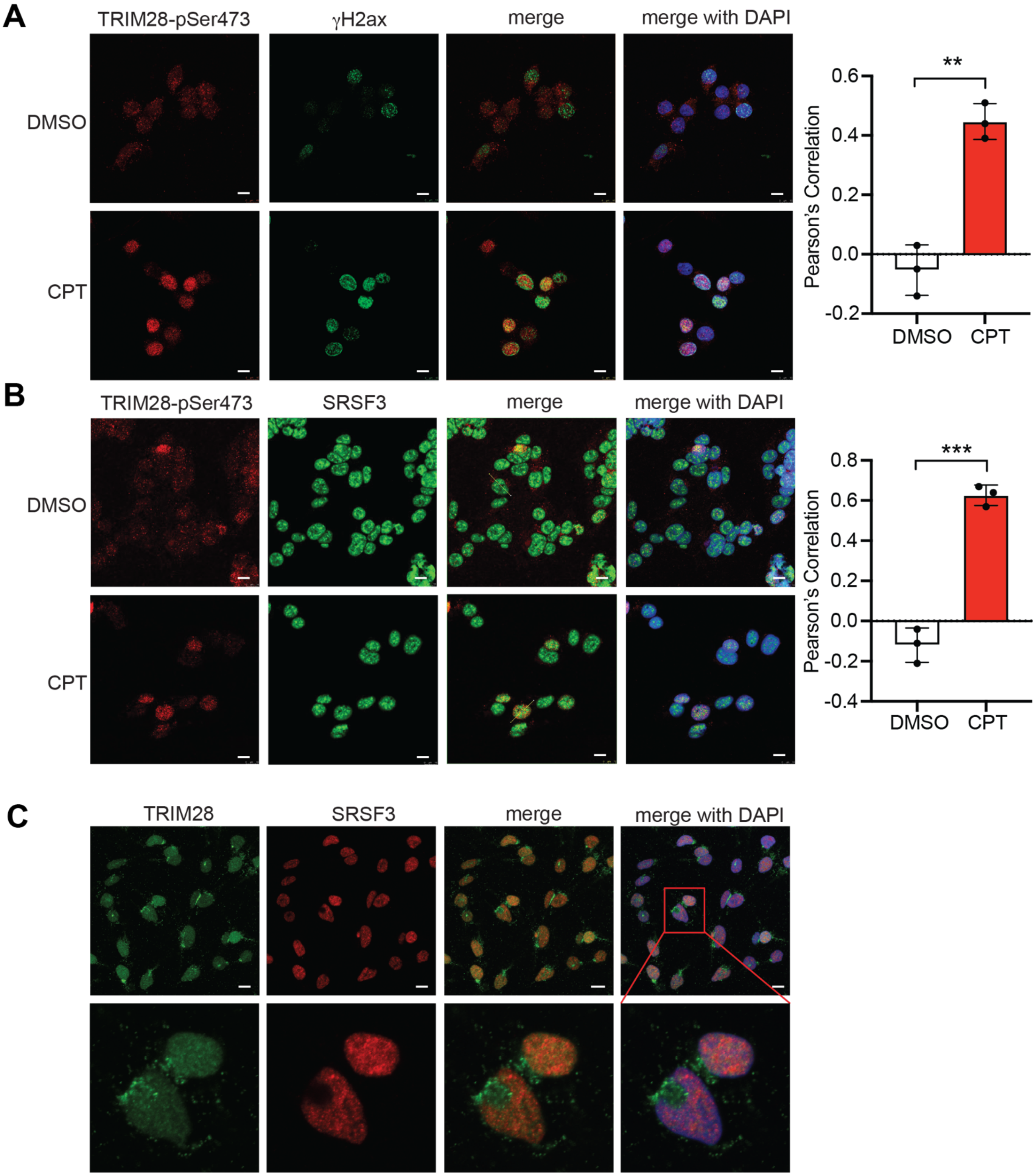

Figure S7: Co-localization of SRSF3 and p-TRIM28(Ser473) to sites of DNA damage. HepG2 cells were treated with DMSO or CPT (5 μM) for 1h. **(A)** Immunofluorescence staining for p-TRIM28(Ser473) and γH2ax. Scale bars: 10 μm. Graph shows Pearson's correlation coefficient measuring the co-localization of two proteins (*n* = 3/group). DMSO (control) group shown in white, CPT group shown in red. **(B)** Immunofluorescence staining for p-TRIM28(Ser473) and SRSF3. Scale bars: 10 μm. Graph shows Pearson's correlation coefficient (*n* = 3/group). DMSO (control) group shown in white, CPT group shown in red. **(C)** Immunofluorescence staining for TRIM28 and SRSF3 in the absence of CPT with enlargement showing spatial separation of TRIM28 and SRSF3. Scale bars: 10 μm. All quantified results are presented as mean ± SD; $**P < 0.01$, $***P < 0.001$ by t-test.
