# Supplementary material for: SRSF3-TRIM28-MDC1 prevents DNA damage caused by R-loops in fatty liver disease in mice
Source: JCI Insight. 2026 Jan 9;11(1):e188629. doi: 10.1172/jci.insight.188629 (PMC12890501; doi:10.1172/jci.insight.188629)

Figure 1A

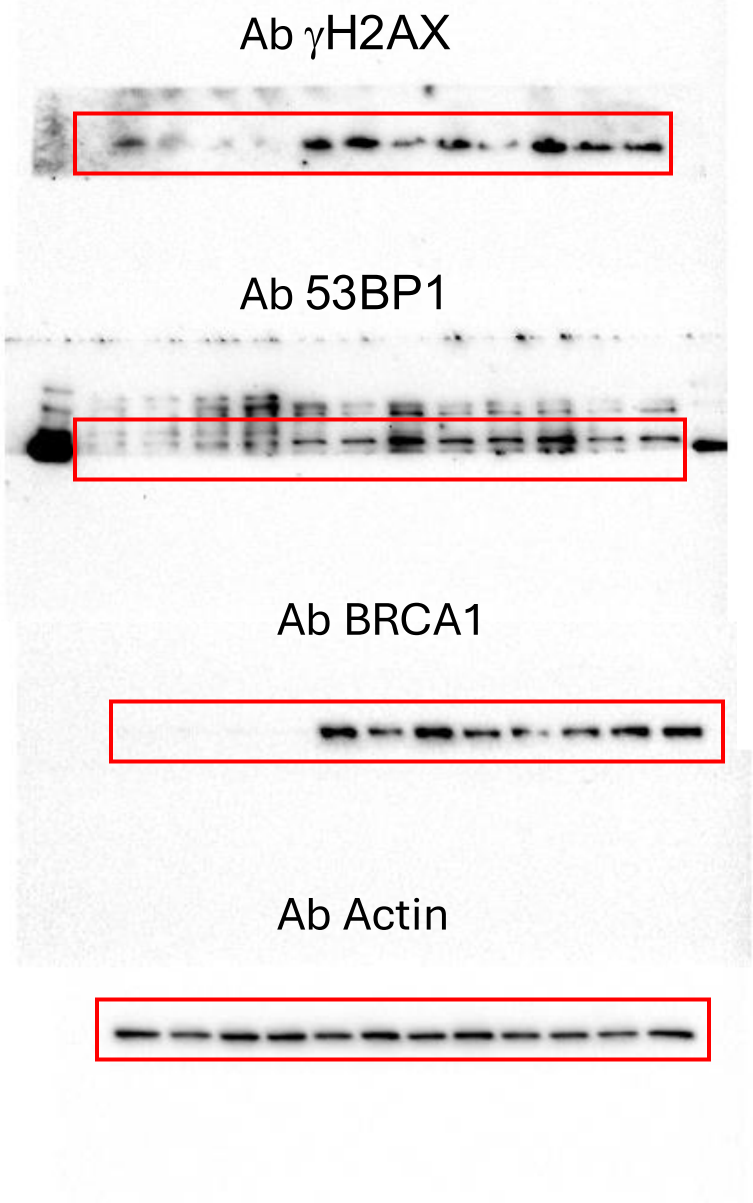

Figure 1A

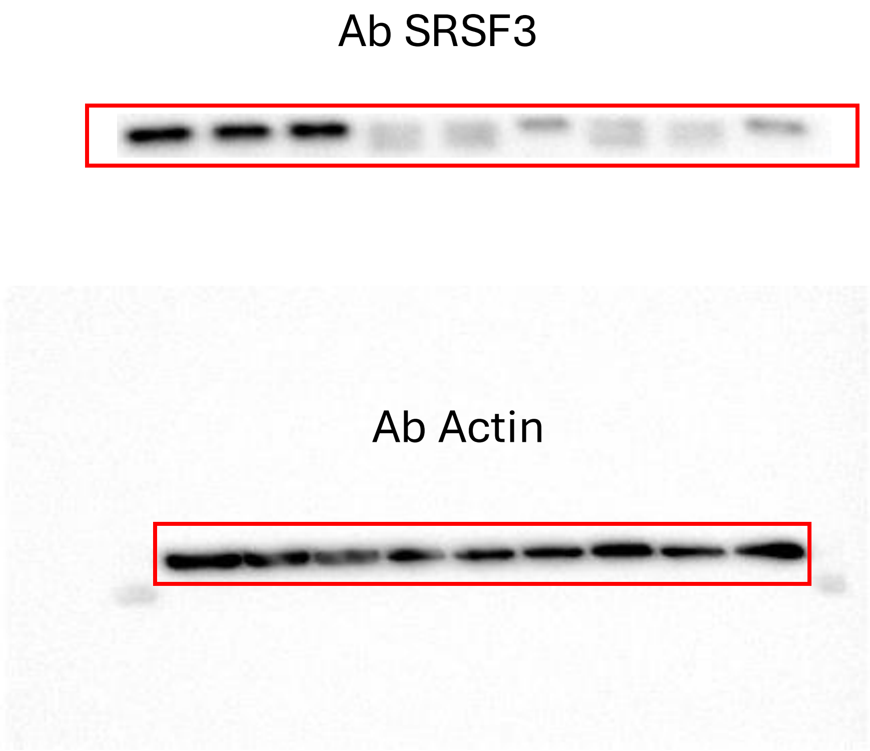

Figure 1B

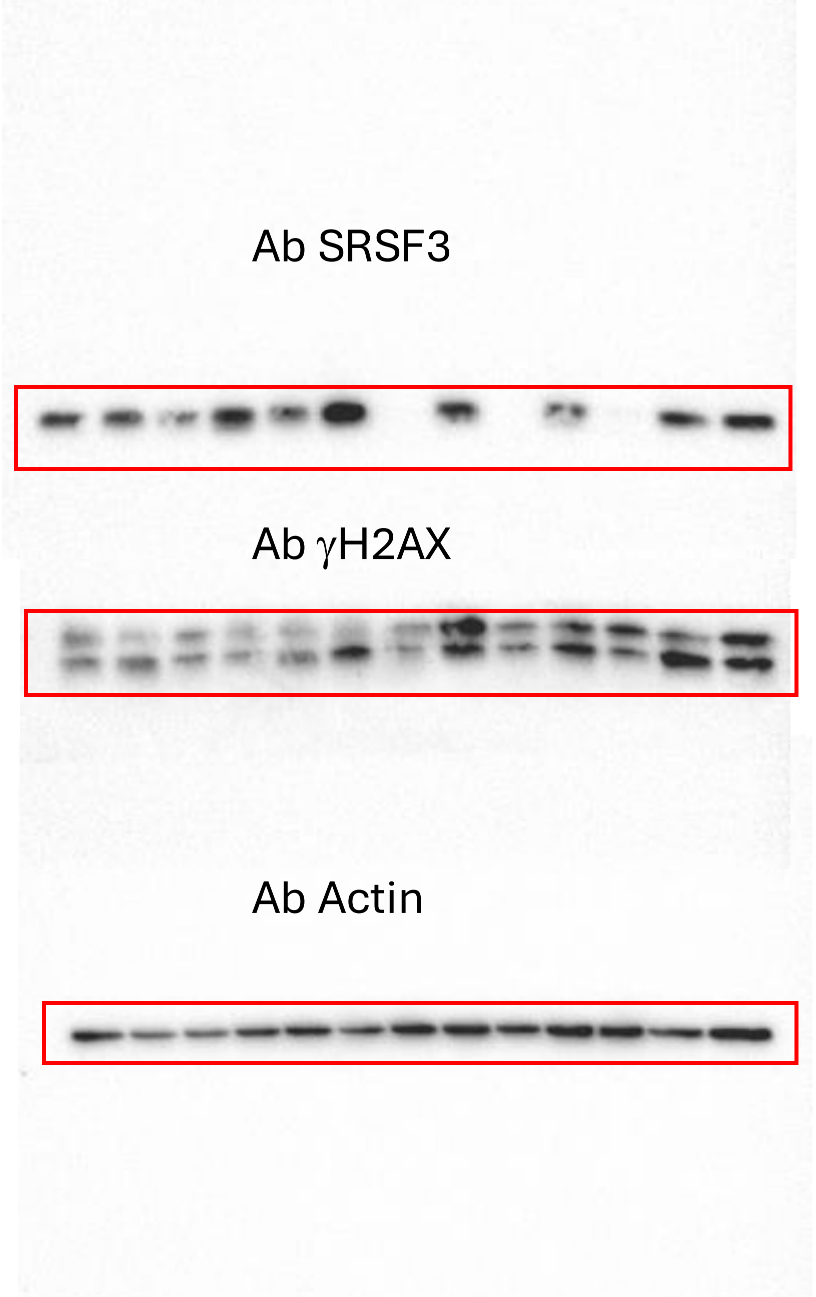

Figure 2B

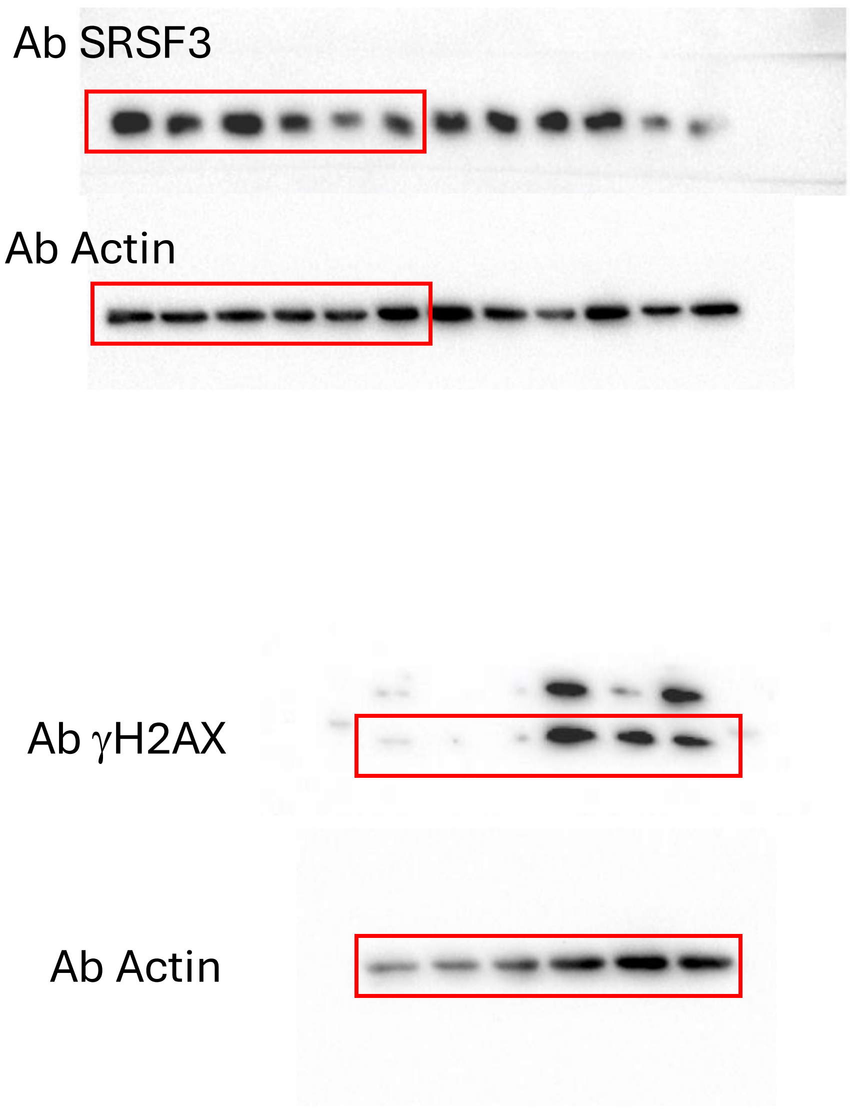

Figure 2B

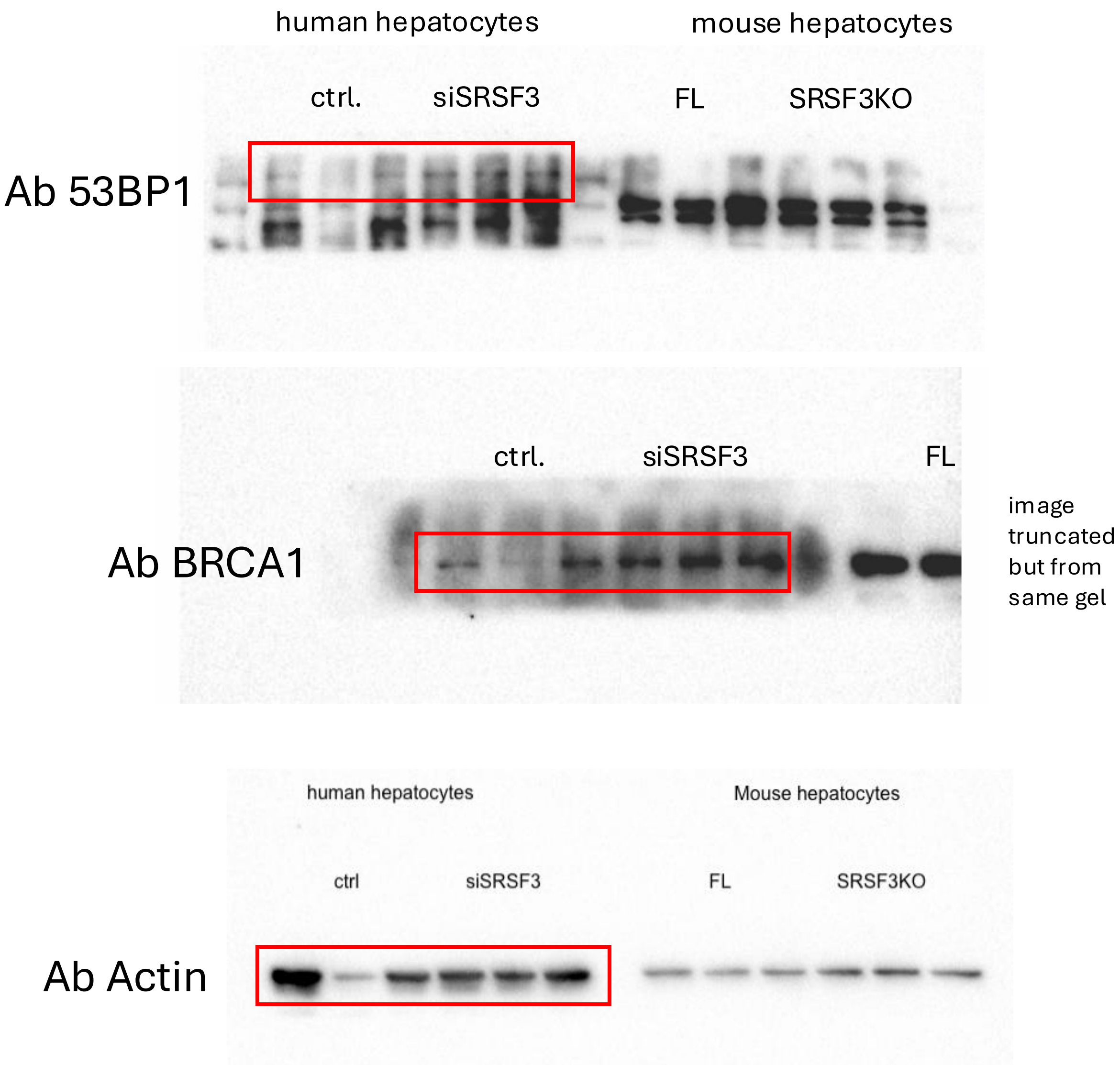

Ab S9.6

Figure 3A

Ab ds DNA

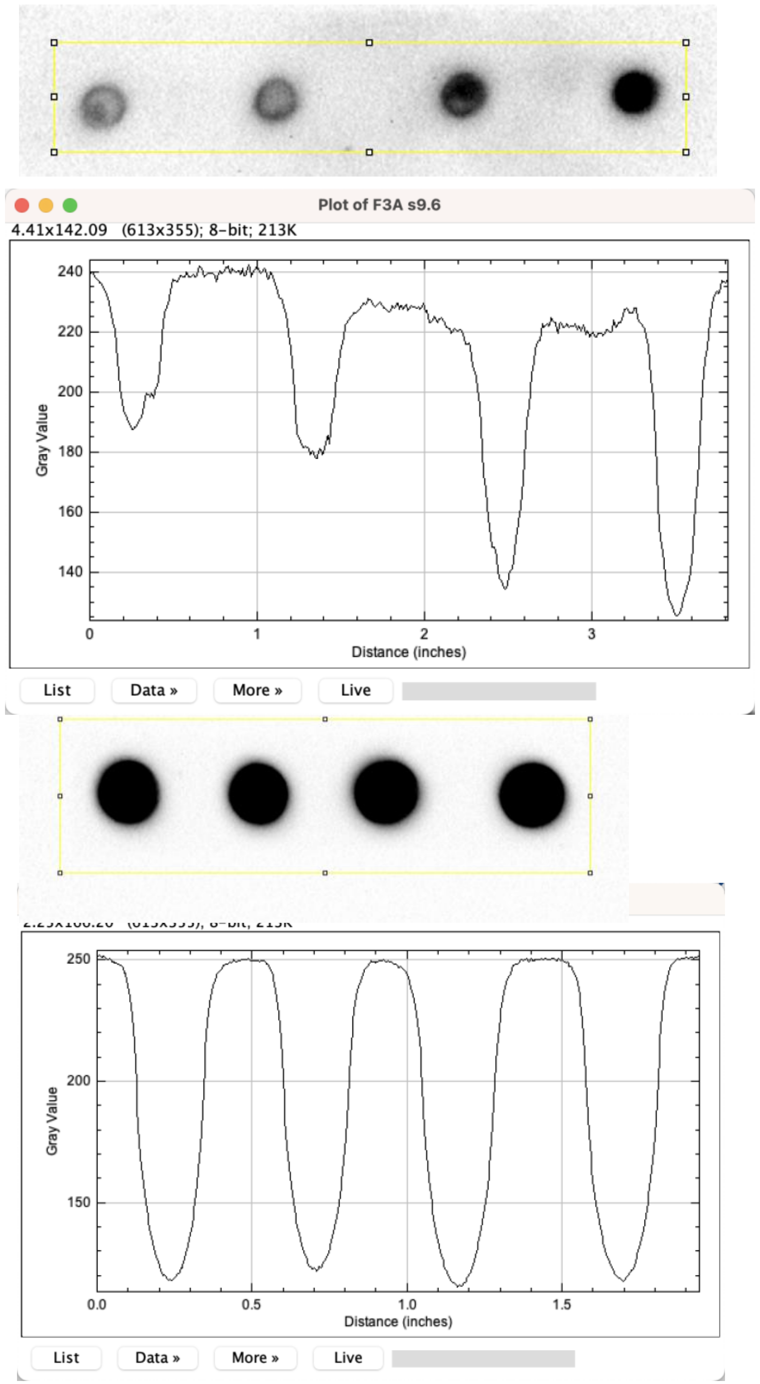

Ab S9.6

Figure 3B

Ab ds DNA

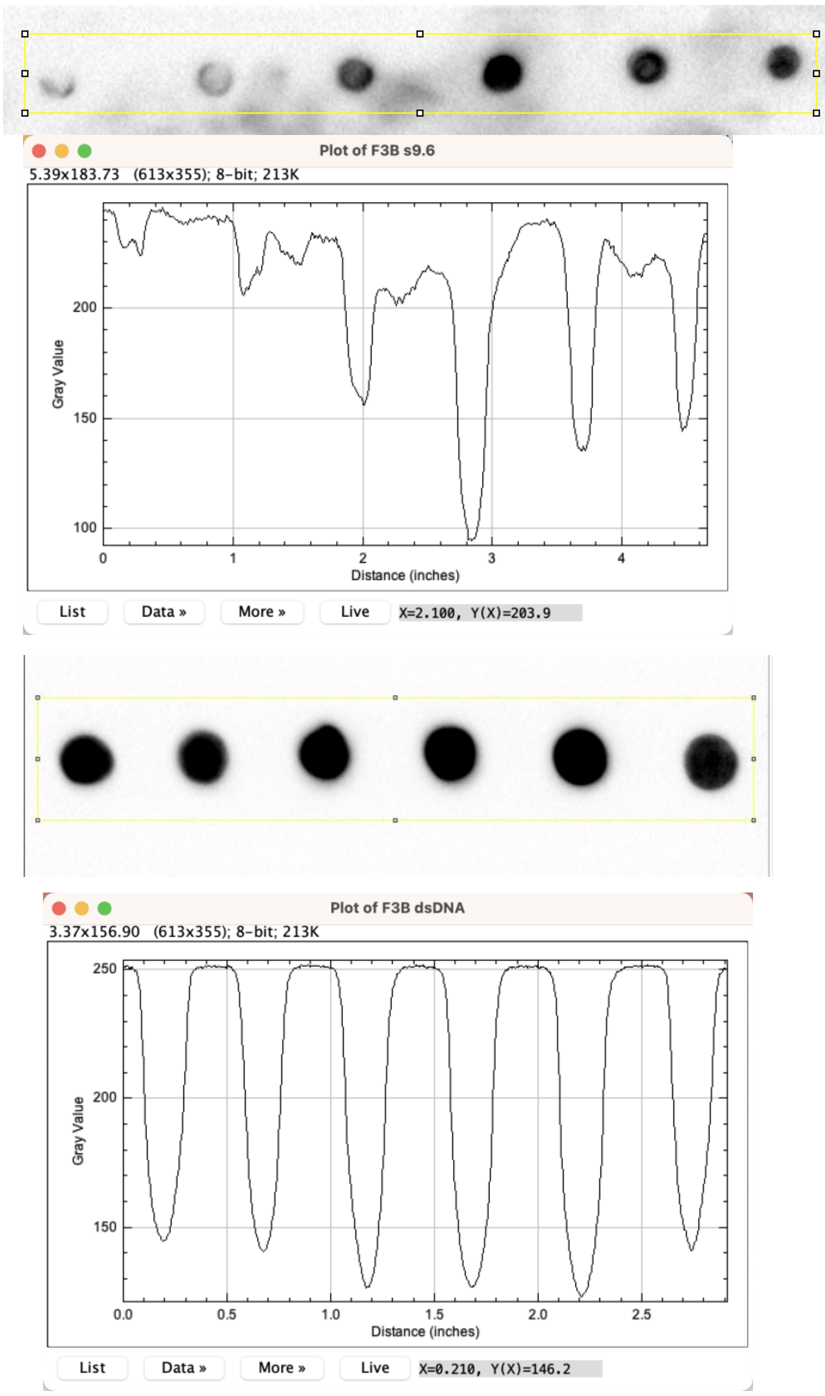

Figure 3C

Ab S9.6

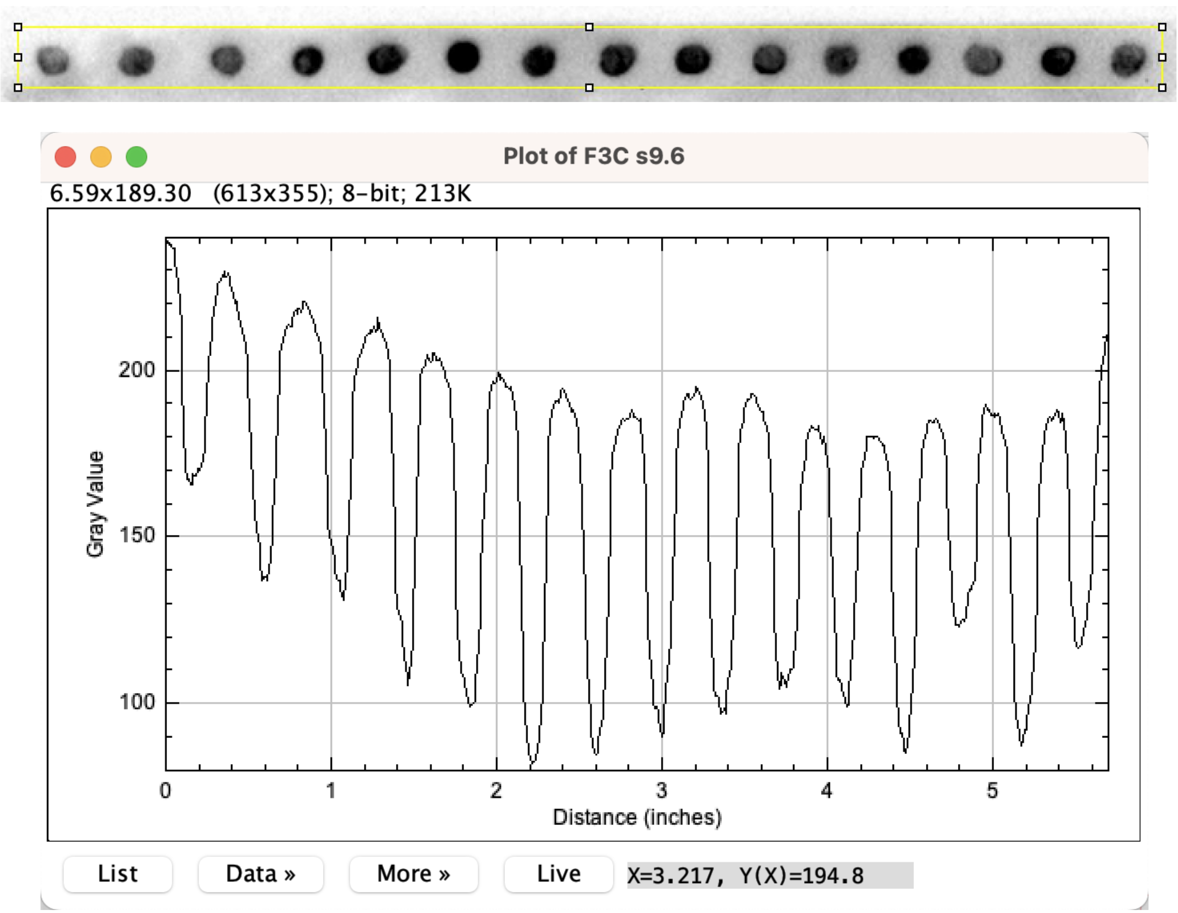

Ab ds DNA

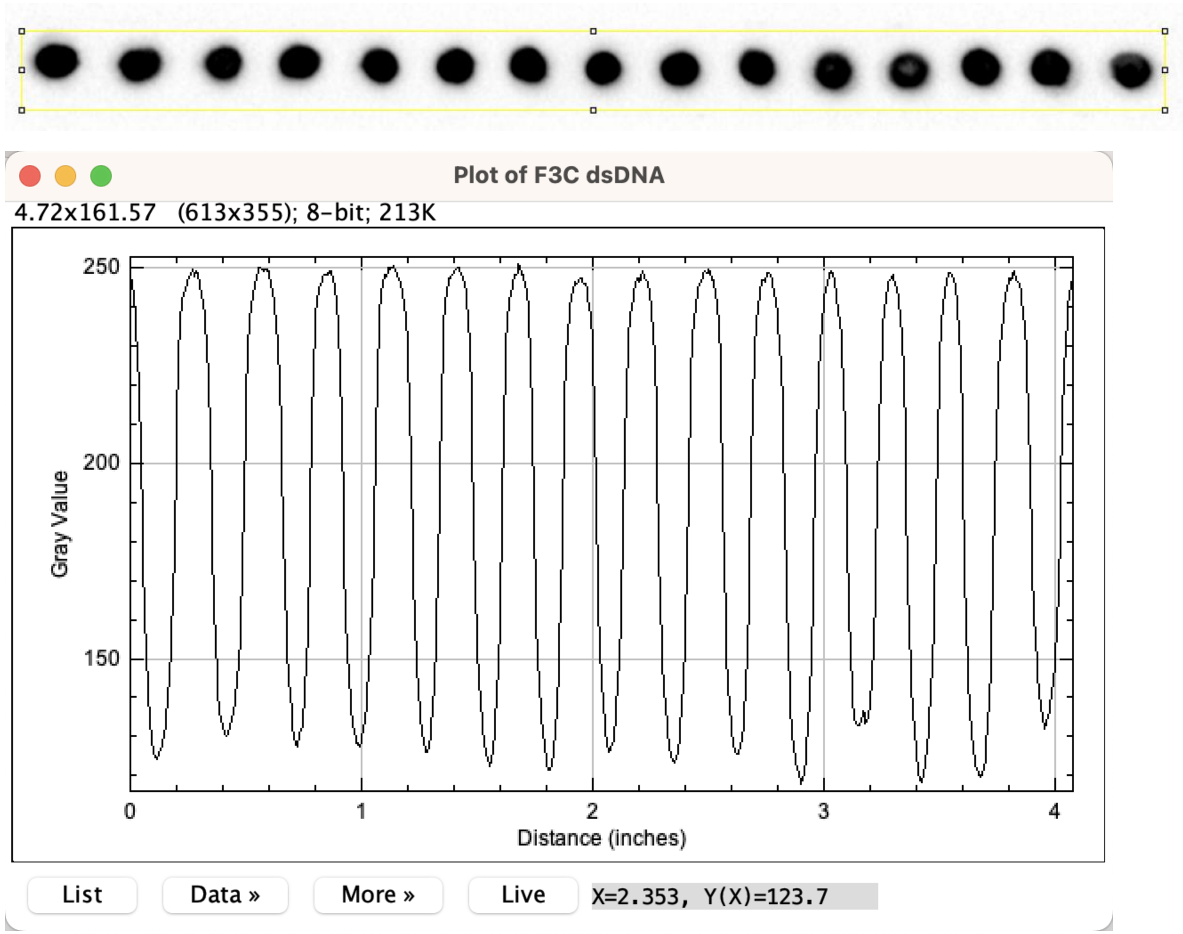

Figure 3D

Ab S9.6

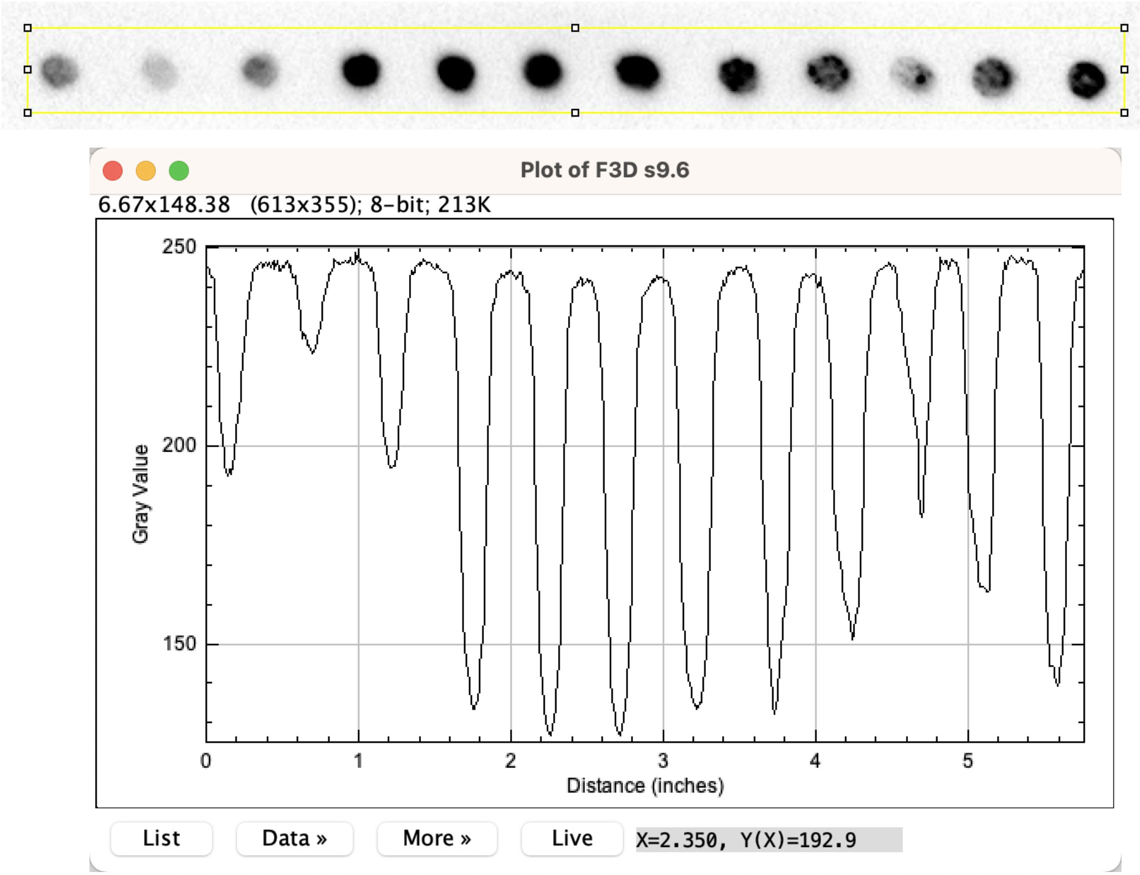

Ab ds DNA

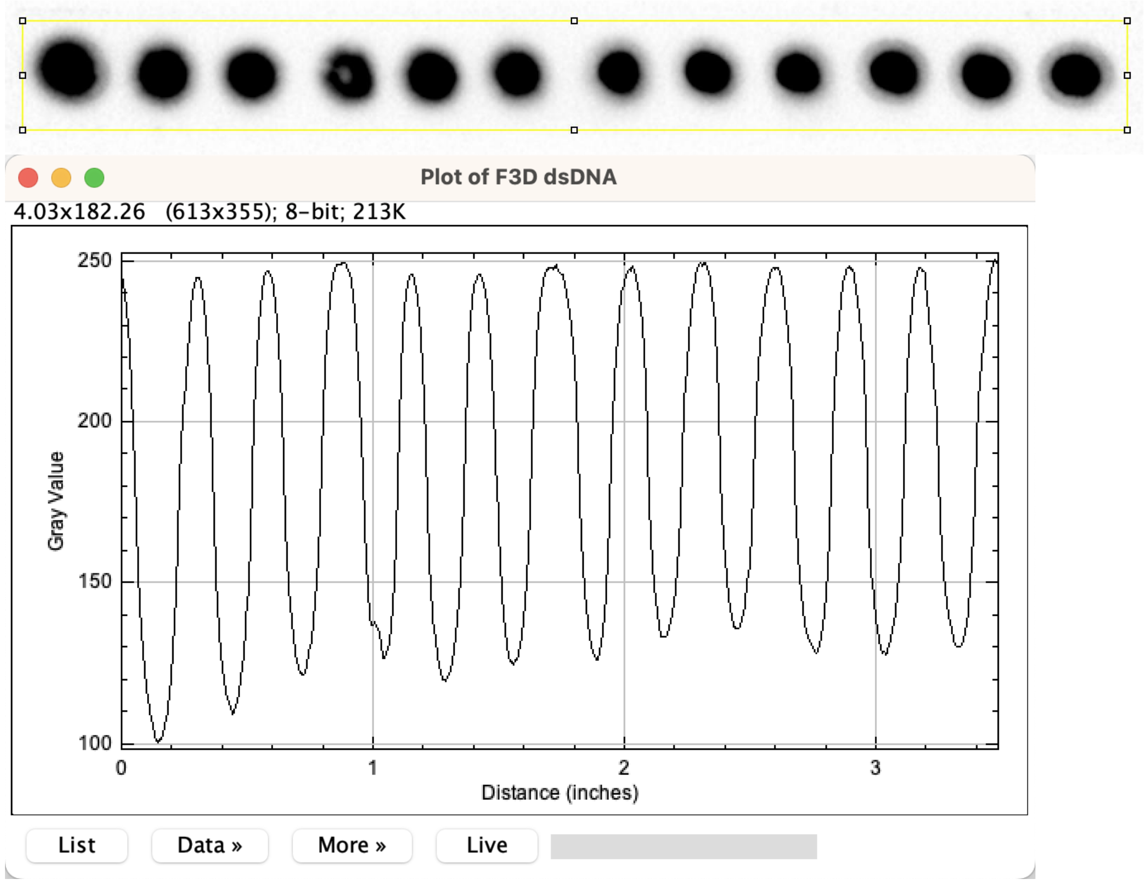

Figure 3E

Ab S9.6

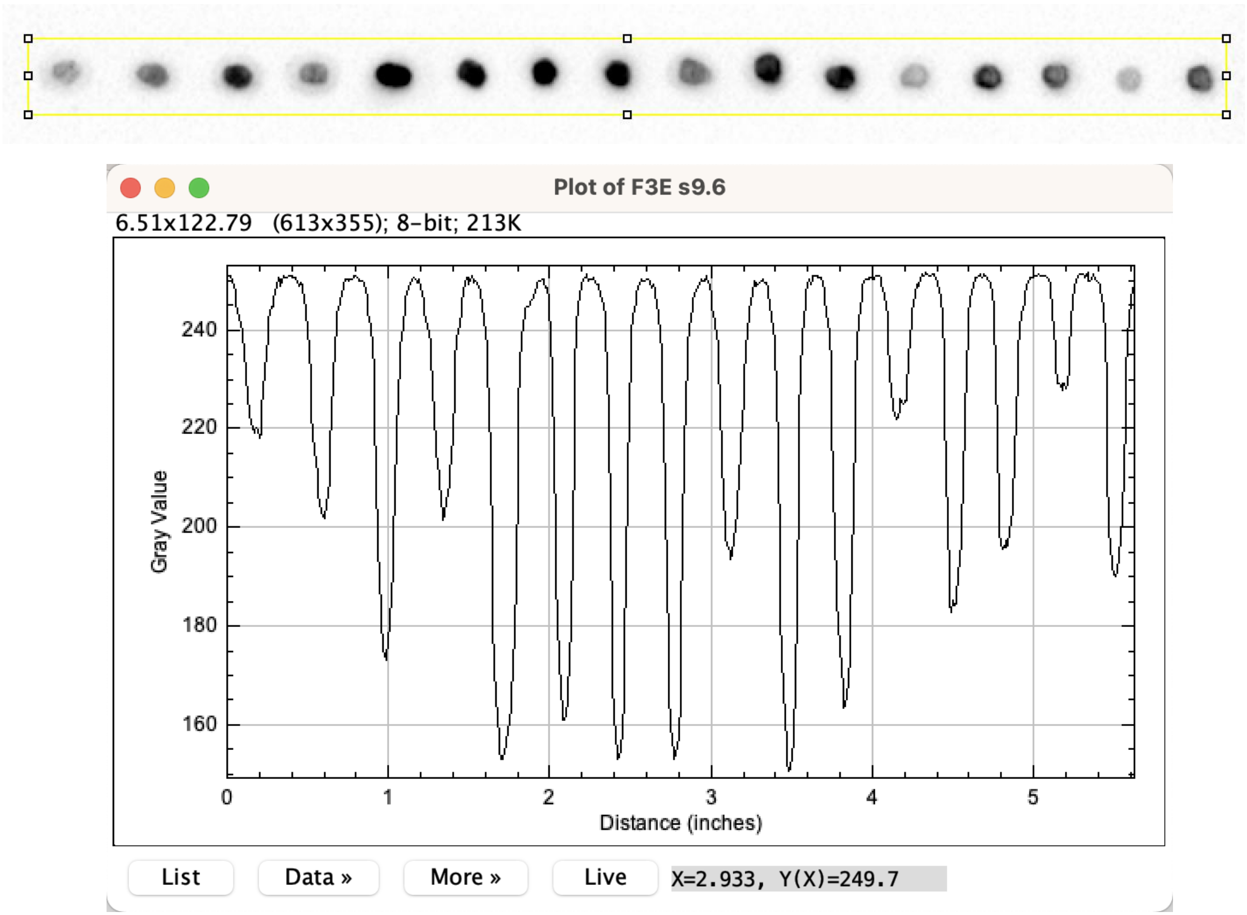

Ab ds DNA

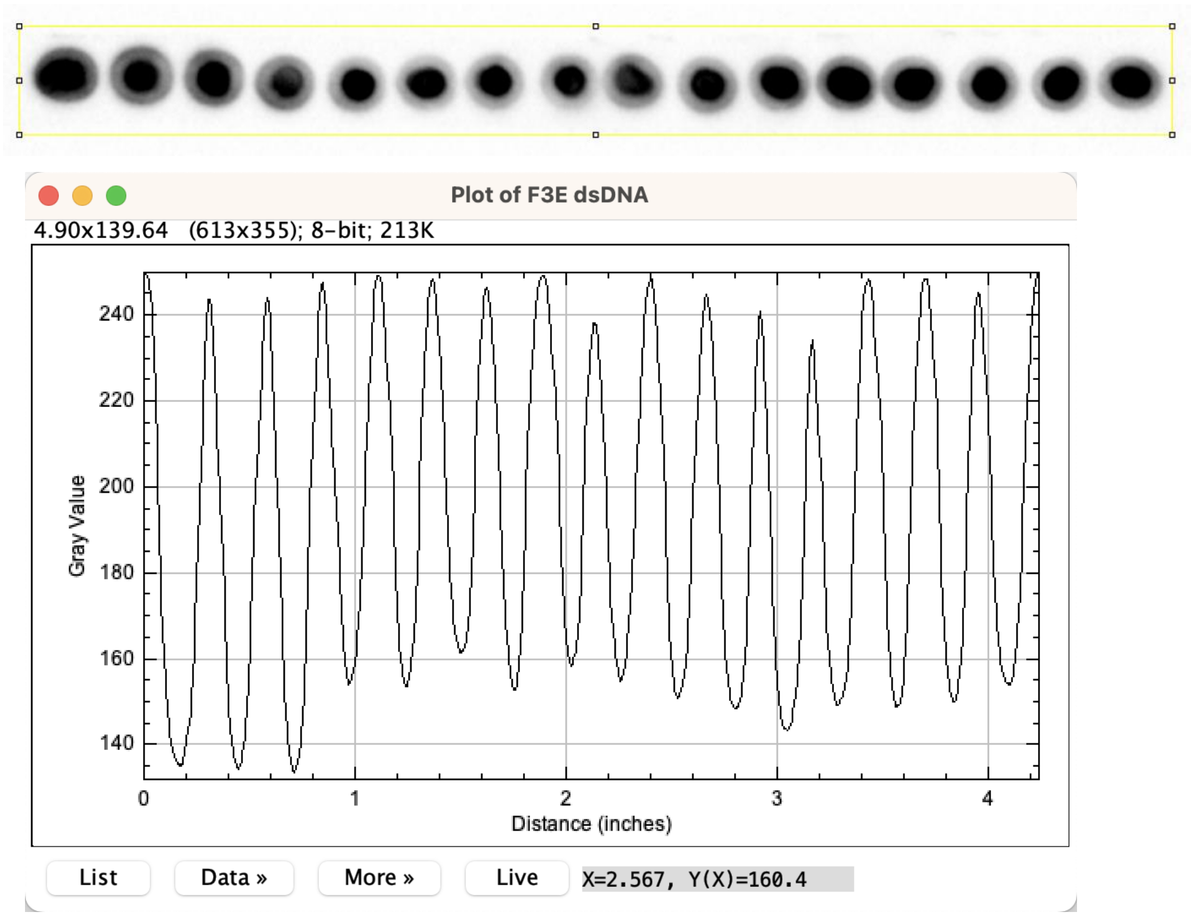

Figure 3F

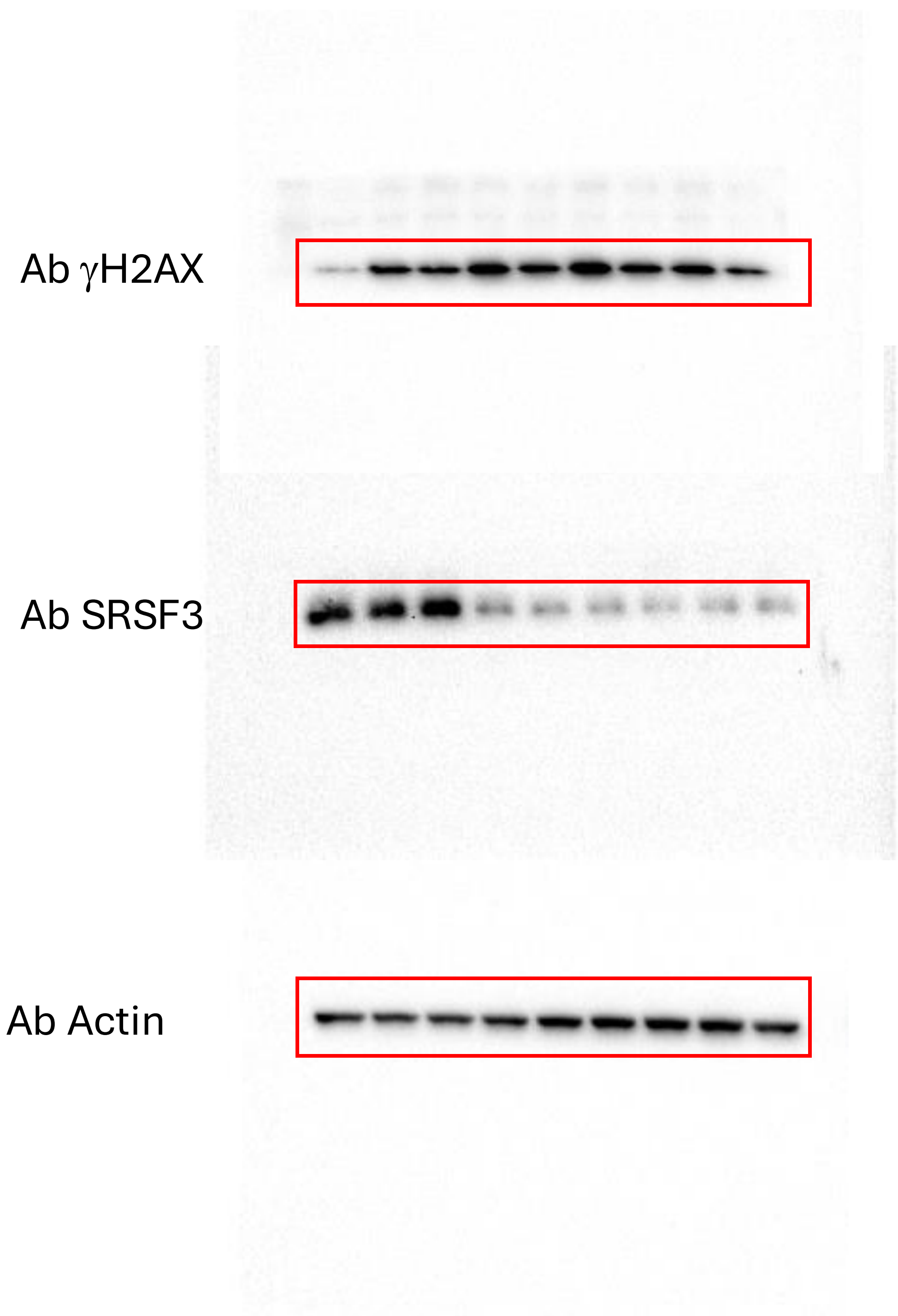

Figure 5C

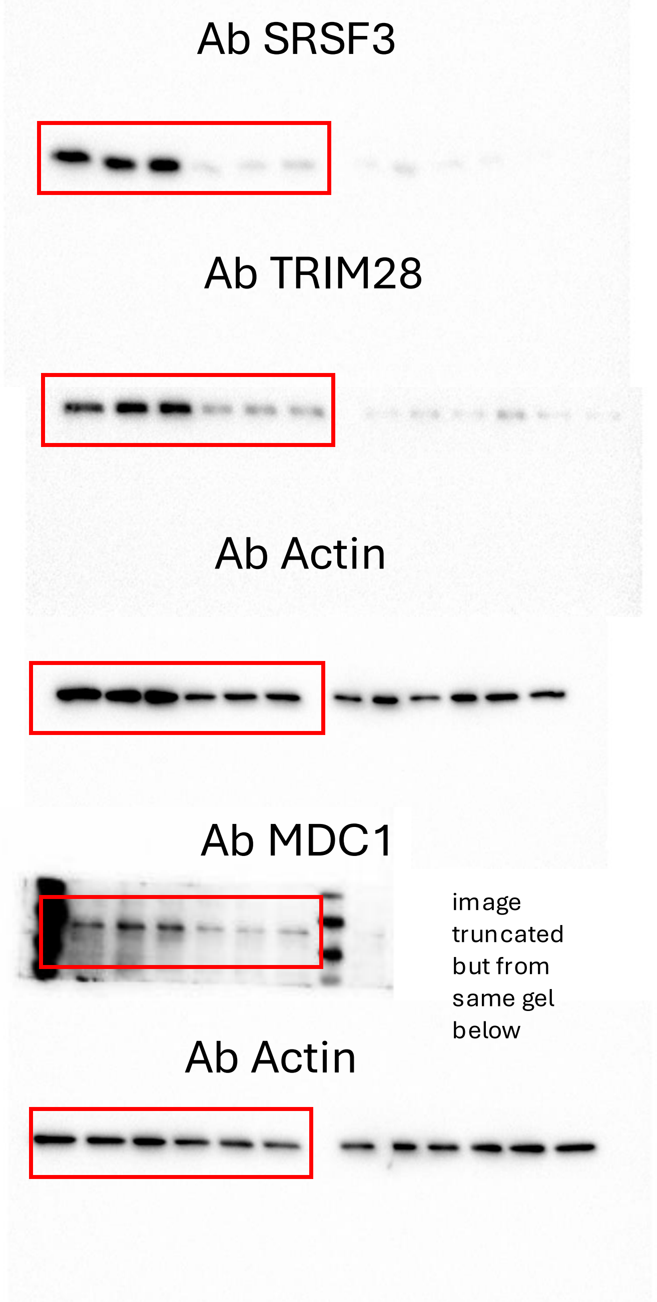

Figure 5D

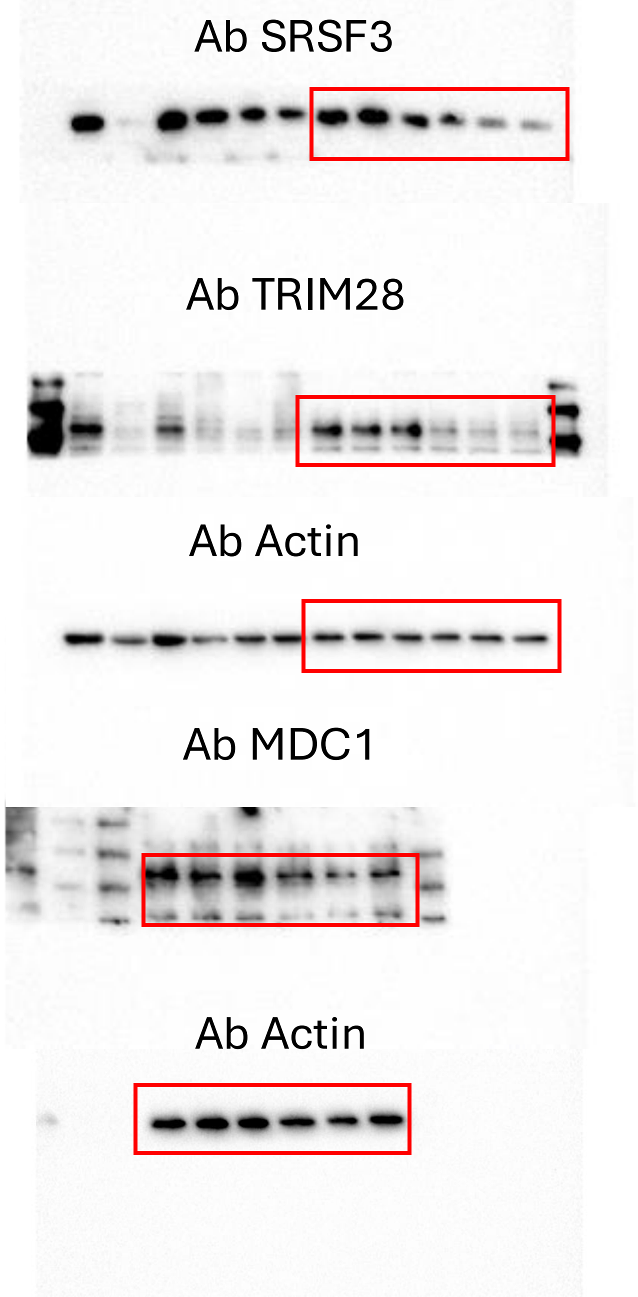

Figure 5E

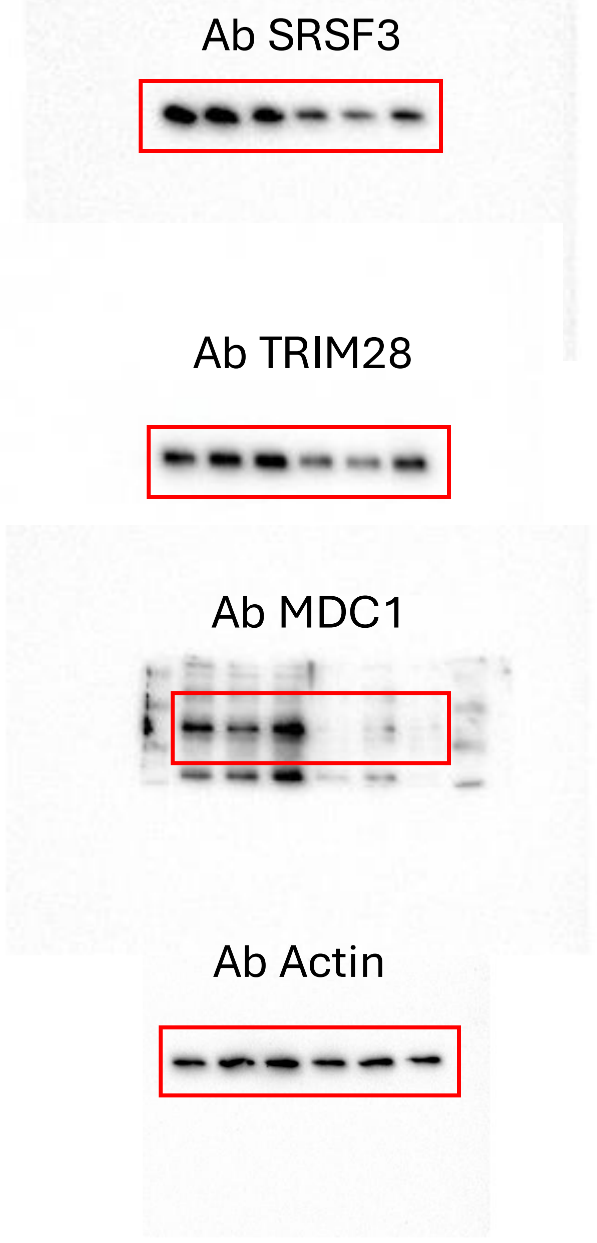

Figure 5F

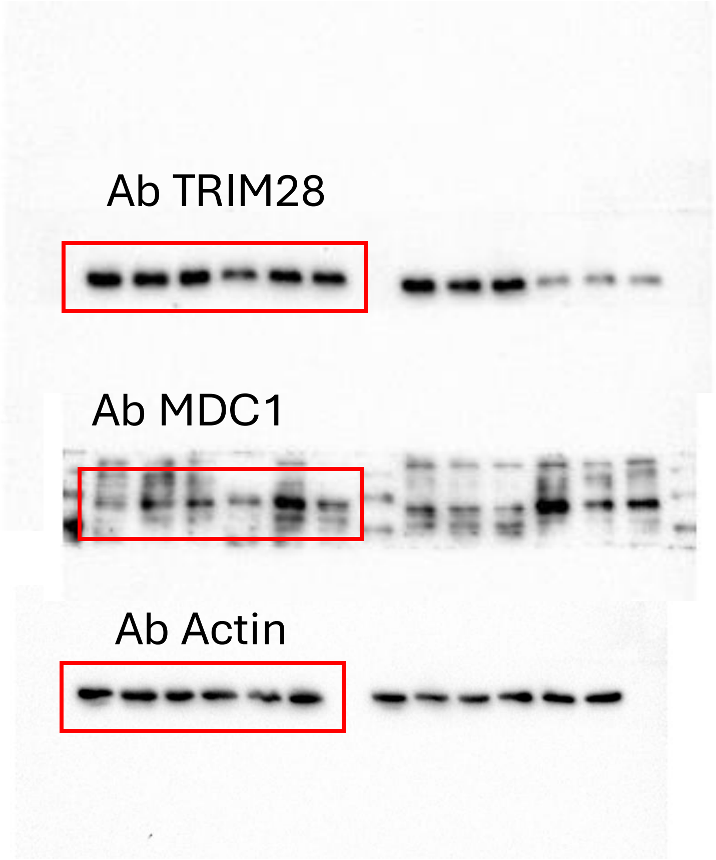

Figure 5G

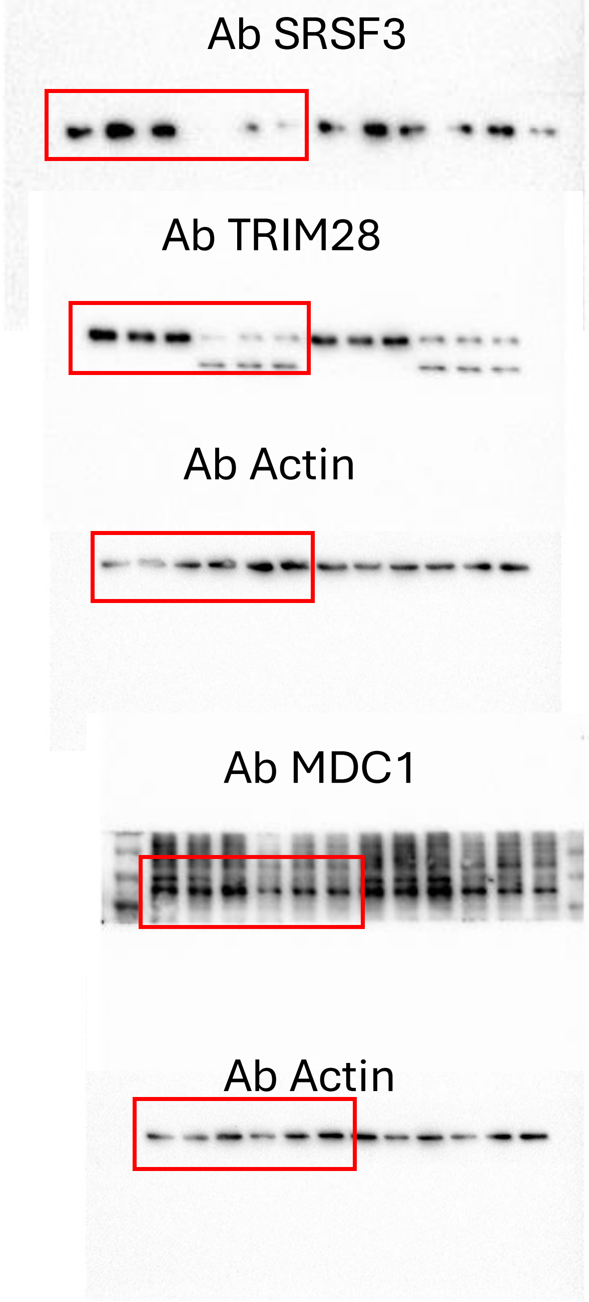

Figure 5H

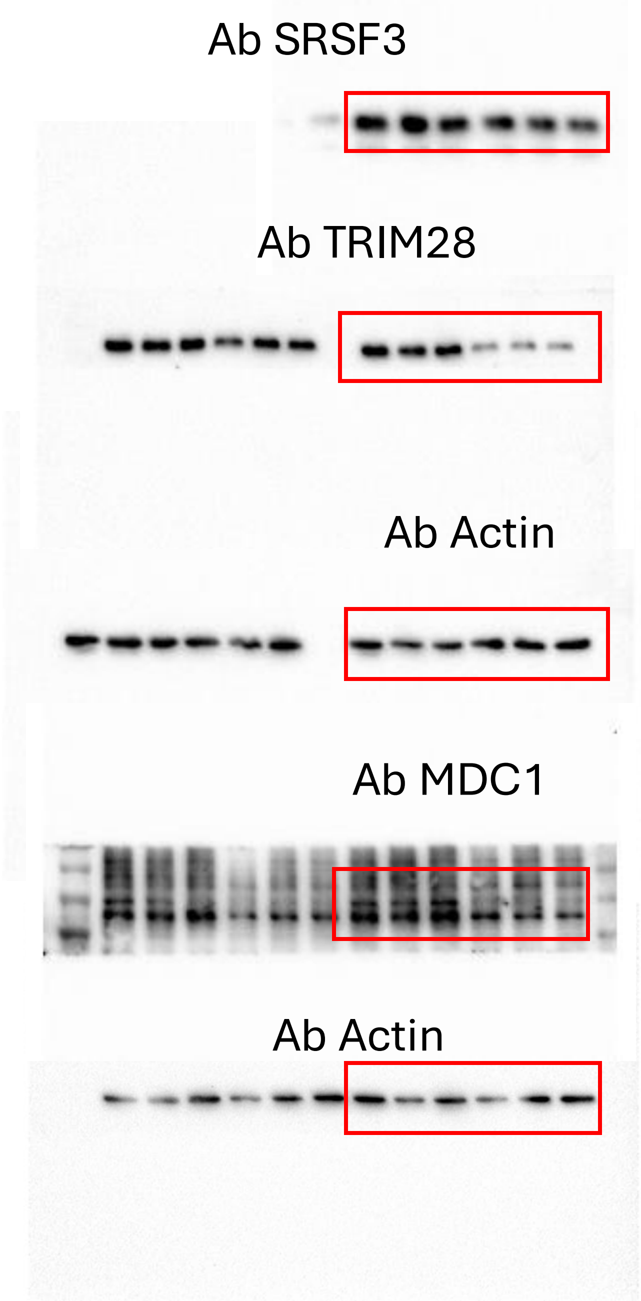

Figure 6A

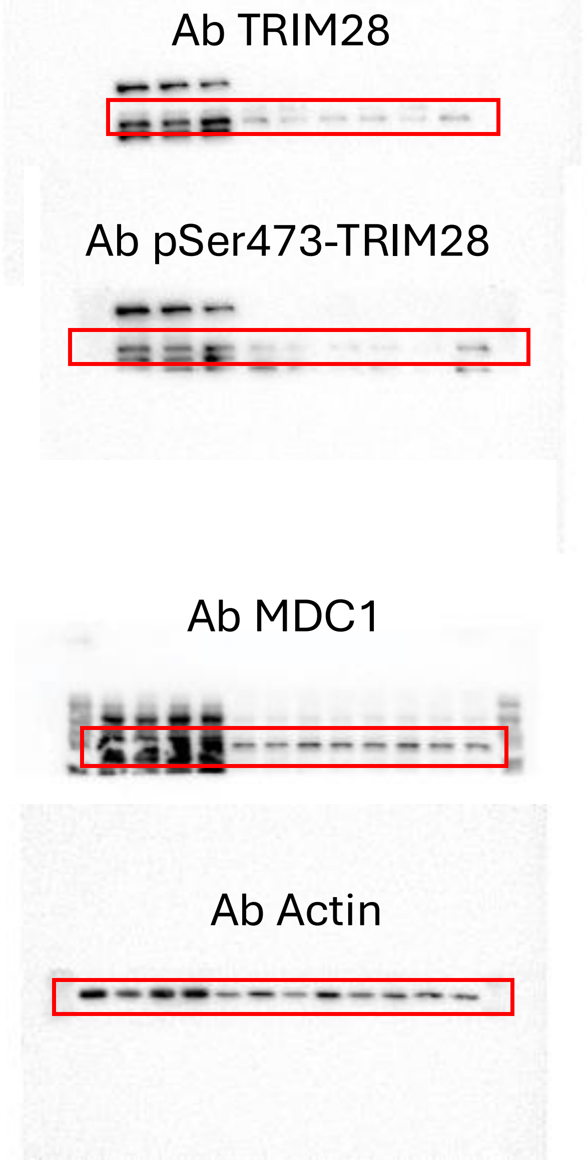

Figure 6B

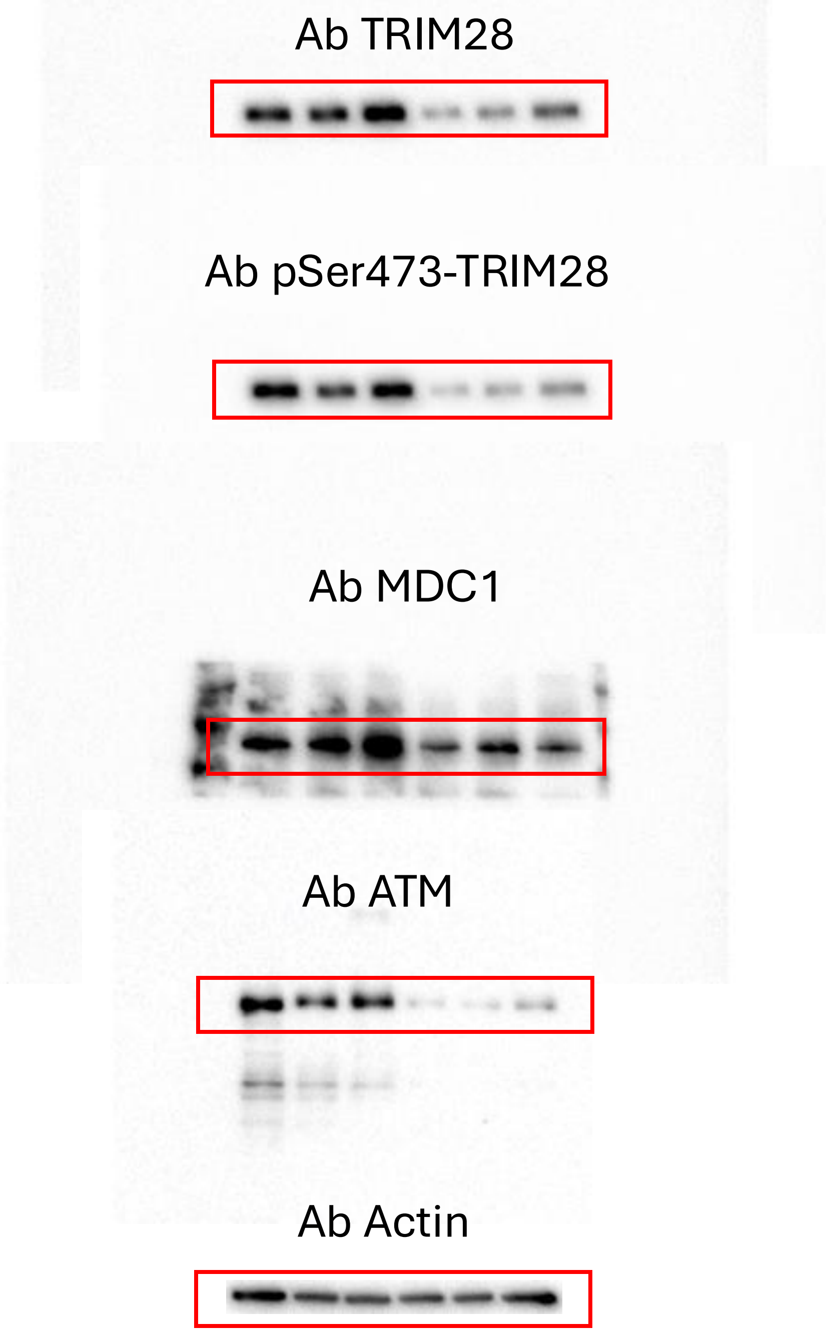

Figure 6C

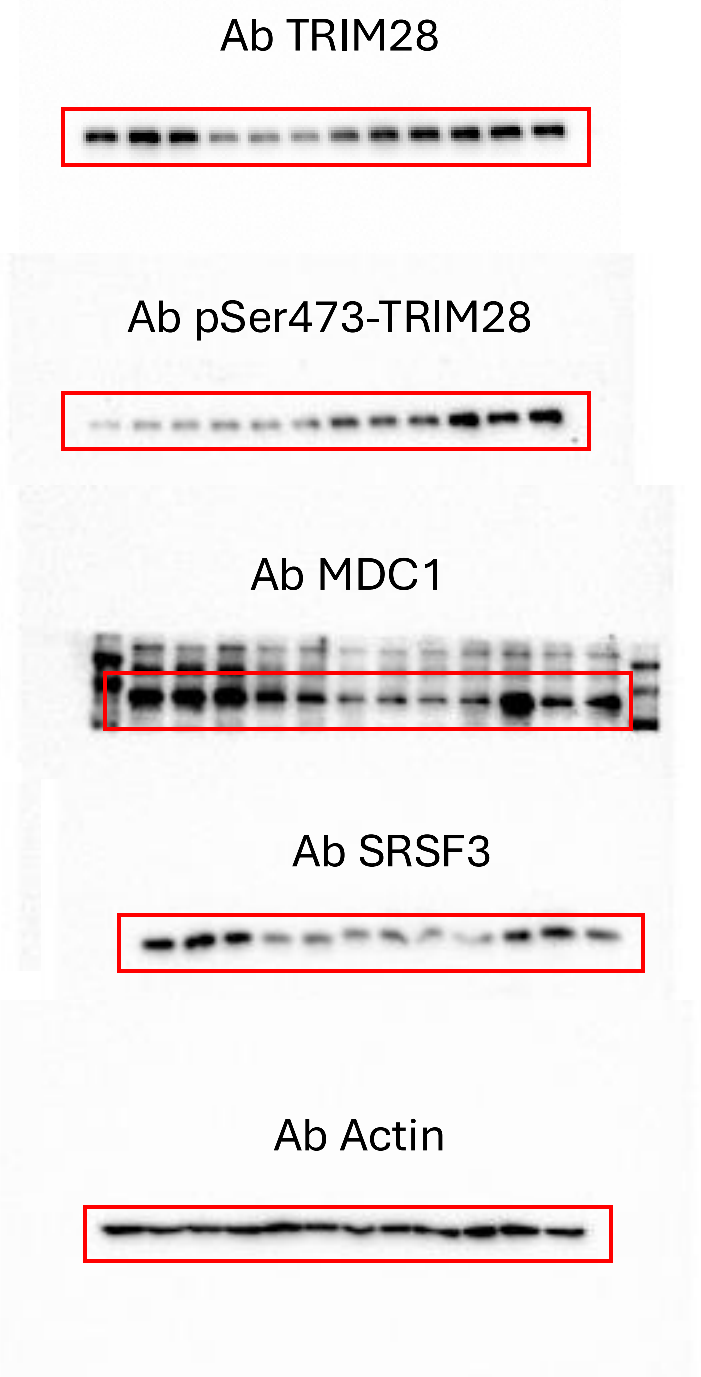

Figure 7A

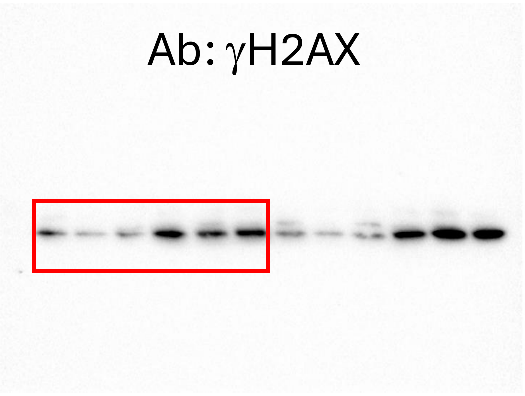

Figure 7B

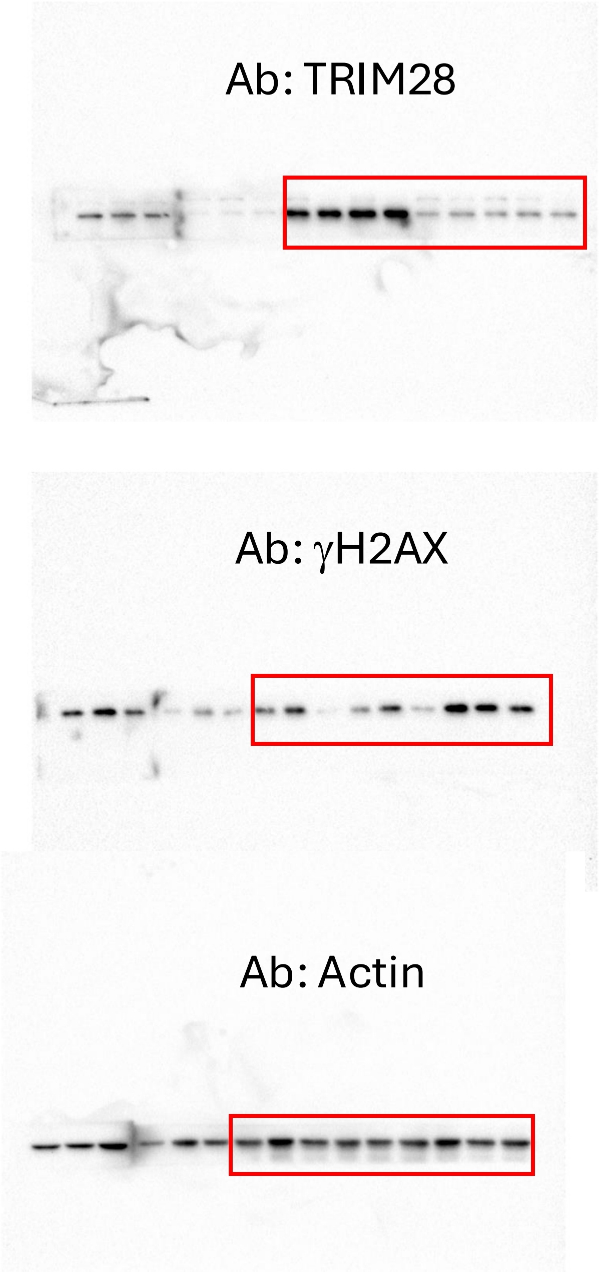

Figure 7C

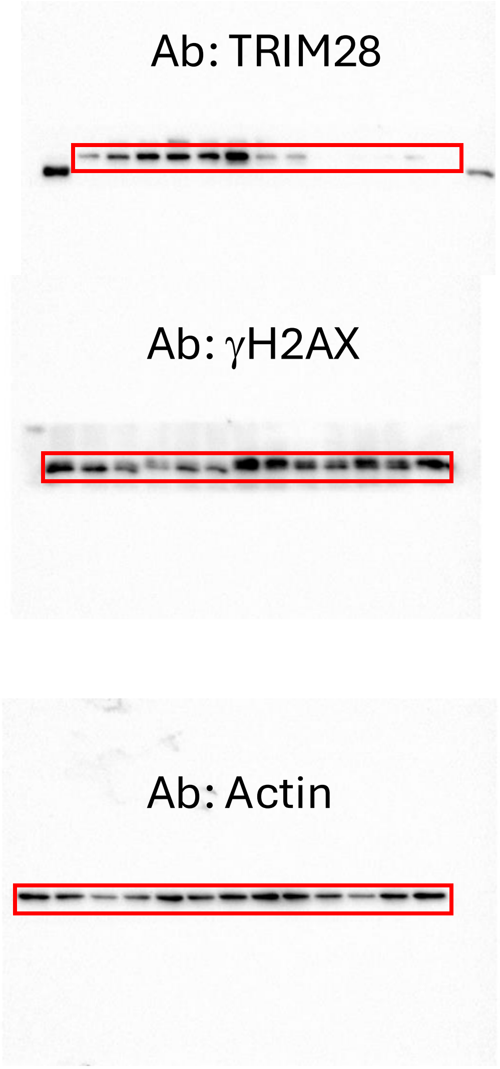

Figure 7D

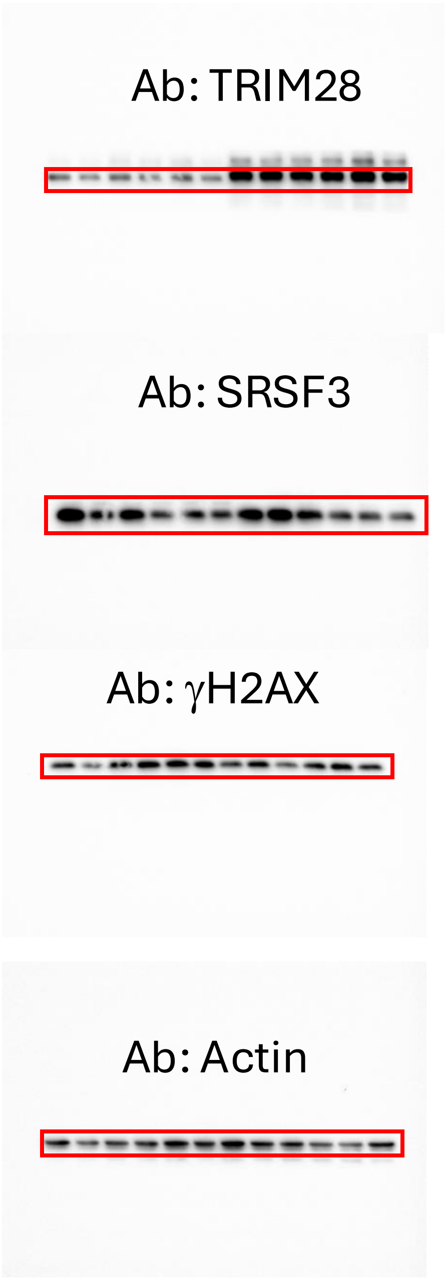

Supplement: Unedited blot and gel images [file jciinsight-11-188629-s018.pdf]
